# Supplementary material for: Wearable in-sensor reservoir computing using optoelectronic polymers with through-space charge-transport characteristics for multi-task learning
Source: Nat Commun. 2023 Jan 28;14:468. doi: 10.1038/s41467-023-36205-9 (PMC9884246; doi:10.1038/s41467-023-36205-9)
Supplement: Supplementary file 1 — Supplementary Information [file 41467_2023_36205_MOESM1_ESM.pdf]

Supplementary Information

Wearable In-Sensor Reservoir Computing Using Optoelectronic Polymers with Through-Space Charge-Transport Characteristics for Multi-Task Learning

Xiaosong Wu[1,#], Shaocong Wang[2,#], Wei Huang[1], Yu Dong[1], Zhongrui Wang*[2] & Weiguo Huang*[1,3,4]

[1]State Key Laboratory of Structural Chemistry, Fujian Institute of Research on the Structure of Matter, Chinese Academy of Sciences, Fuzhou, Fujian 350002, P. R. China.

[2]Department of Electrical and Electronic Engineering, University of Hong Kong, Pokfulam Road, Hong Kong SAR

[3]Fujian Science & Technology Innovation Laboratory for Optoelectronic Information of China, Fuzhou, Fujian 350002, P. R. China.

[4]University of Chinese Academy of Sciences, 19A Yuquan Road, Beijing 100049, P. R. China.

[#]These authors contributed equally: Xiaosong Wu, Shaocong Wang.

Table of Contents

1. Experimental Section……………………….………………………………S-2

1.1 Measurement and Characterization …………..…………………………S-2

2. Device Fabrication Details……………………………..……………………S-3

2.1 *p*-NDI Based Transistor …………………………..…..………………S-3

2.2 C8-NDI Based Transistor …………...………………..…………………S-3

2.3 C8-NDI-C12 Based Transistor …………...………………...……………S-4

2.4 Pentacene Based Transistor …………...……………..…………………..S-4

2.5 P3HT Based Transistor …………...………………..……………………S-5

2.6 C8-NDI/*p*-NDI based transistor..S-5

2.7 Electrical Characterization S-6

2.8 Mobility Calculation S-6

2.9 HOMO and LUMO Calculation S-6

3. Simulations of the Reservoir Computing..S-7

3.1 Workflow of the Reservoir S-7

3.2 Simulations of the Readout Map Training S-7

3.3 Software Baseline S-8

4. Supporting Figures..S-8

5. Synthesis of Materials..S-25

5.1 Synthesis of Semiconducting Polymer (*p*-NDI) S-25

5.2 Synthesis of Semiconducting Polymer (C8-NDI) S-26

5.3 Synthesis of PolyAT and PolyES S-27

1. Experimental Section

Measurement and Characterization: NMR measurements were performed on a Bruker AVANCE III NMR 400M instruments. ATR tests were conducted on a Bruker VERTEX 70 spectrometer. The UV-vis absorption spectra were determined by Shimadzu UV-2600 UV-vis spectrophotometer. Fluorescence emission spectra and fluorescence lifetimes were measured by Edinburgh FLS1000 Spectrometer. Thermogravimetric Analysis (TGA) measurements were tested on a Netzsch STA449F3 analyzer at a heating rate of 10 °C/min, Differential scanning calorimetry (DSC) measurements were tested on Perkin-Elmer Diamond DSC instrument, under a nitrogen atmosphere at a heating/cooling rate of 10 °C/min. Atomic force microscopy (AFM) measurements were performed under ambient conditions using a Digital Instrument Multimode Nanoscope IIIA in the tapping mode. The 2D GIWAXS patterns were acquired using a XEUSS SAXS/WAXS system at the National Center for

Nanoscience and Technology (NCNST, Beijing). High resolution transmission electron microscopy (TEM) images were performed on a FEI-Talos F200X, and scanning electron microscope (SEM) characteristics were performed on Zeiss FE-SEM Sigma300. The film thicknesses were measured by a Bruker DEKTAK XT profile meter.

2. Device Fabrication Details

***p*-NDI Based Transistor:** A solution containing methyl methacrylate (MMA), hexamethylene diacrylate (20% in molar ratio with respect to MMA), 0.01% phenylbis(2,4,6-trimethylbenzoyl)phosphine oxide and 0.07 mL toluene was drop casted on ITO-coated PEN substrate and photocured in N_2. The resulting PMMA dielectric layer (250 nm) was annealed at 100°C for 1 h in N_2. *p*-NDI (10 mg/mL, $CHCl_3$) was spin-casted on PMMA layer and annealed at 100°C for 1 h in N_2 to form a 32 ± 2 nm semiconducting layer. Finally, 50 nm Au source and drain electrodes were thermally evaporated on the semiconducting layer through a shadow mask at a rate of 0.5 Å/s. The W and L of the channels are 5600 μm and 200 μm, respectively. For transistors used for signal sensing, memorizing, preprocessing, and optoelectronic in-sensor RC**,** the dimension of the Au electrodes is 630 × 630 μm.

For *p*-NDI/SiO_2 based transistor, 32 ± 2 nm *p*-NDI was spin-coated on n^{++} Si wafer with 300 nm SiO_2 (with/without HMDS treatment, HMDS: Hexamethyldisilane) and annealed at 100 °C for 1 h in N_2 to form a semiconducting layer. Finally, 50 nm Au source and drain electrodes were thermally evaporated on the semiconducting layer through a shadow mask at a rate of 0.5 Å/s. The W and L of the channels are 5600 μm and 200 μm, respectively.

C8-NDI Based Transistor: PMMA (250 nm) was spin-coated on ITO-coated PEN substrate. The resulting PMMA dielectric layer was annealed at 100 °C for 1 h in N_2. C8-NDI (triple sublimed) was then thermally evaporated directly onto PMMA dielectric layer with a thickness of 50 nm at a rate of 0.5 Å s^{-1}, substrate temperature during the deposition was held constant at 30°C. The deposition chamber pressure was $< 5 \times 10^{-6}$ Torr. Finally, 50 nm Au source and drain electrodes were thermally

evaporated on the semiconducting layer through a shadow mask at a rate of 0.5 Å/s. The *W* and *L* of the channels are 5600 μm and 200 μm, respectively.

For C8-NDI/SiO_2 based transistor: C8-NDI (triple sublimed) was thermally evaporated directly onto n^{++} Si wafer bearing 300 nm SiO_2 dielectric layer with a thickness of 50 nm at a rate of 0.5 Å s^{-1}, substrate temperature during the deposition was held constant at 30°C. The deposition chamber pressure was $< 5 \times 10^{-6}$ Torr. Finally, 50 nm Au source and drain electrodes were thermally evaporated on the semiconducting layer through a shadow mask at a rate of 0.5 Å/s. The *W* and *L* of the channels are 5600 μm and 200 μm, respectively.

C8-NDI-C12 Based Transistor: PMMA (250 nm) was spin-coated on ITO-coated PEN substrate. The resulting PMMA dielectric layer was annealed at 100 °C for 1 h in N_2. C8-NDI-C12 (triple sublimed) was then thermally evaporated directly onto PMMA dielectric layer with a thickness of 50 nm at a rate of 0.5 Å s^{-1}, substrate temperature during the deposition was held constant at 30°C. The deposition chamber pressure was $< 5 \times 10^{-6}$ Torr. Finally, 50 nm Au source and drain electrodes were thermally evaporated on the semiconducting layer through a shadow mask at a rate of 0.5 Å/s. The *W* and *L* of the channels are 5600 μm and 200 μm, respectively.

For C8-NDI-C12/SiO_2 based transistor: C8-NDI-C12 (triple sublimed) was thermally evaporated directly onto n^{++} Si wafer bearing 300 nm SiO_2 dielectric layer with a thickness of 50 nm at a rate of 0.5 Å s^{-1}, substrate temperature during the deposition was held constant at 30°C. The deposition chamber pressure was $< 5 \times 10^{-6}$ Torr. Finally, 50 nm Au source and drain electrodes were thermally evaporated on the semiconducting layer through a shadow mask at a rate of 0.5 Å/s. The *W* and *L* of the channels are 5600 μm and 200 μm, respectively.

Pentacene Based Transistor: PMMA (250 nm) was spin-coated on ITO-coated PEN substrate. The resulting PMMA dielectric layer was annealed at 100 °C for 1 h in N_2. Pentacene (triple sublimed) was then thermally evaporated directly onto PMMA dielectric layer with a thickness of 50 nm at a rate of 0.5 Å s^{-1}, substrate temperature

during the deposition was held constant at 30°C. The deposition chamber pressure was $< 5 \times 10^{-6}$ Torr. Finally, 50 nm Au source and drain electrodes were thermally evaporated on the semiconducting layer through a shadow mask at a rate of 0.5 Å/s. The *W* and *L* of the channels are 5600 μm and 200 μm, respectively.

For pentacene/SiO_2 based transistor: Pentacene (triple sublimed) was thermally evaporated directly onto n^{++} Si wafer bearing 300 nm SiO_2 dielectric layer with a thickness of 50 nm at a rate of 0.5 Å s^{-1}, substrate temperature during the deposition was held constant at 30°C. The deposition chamber pressure was $< 5 \times 10^{-6}$ Torr. Finally, 50 nm Au source and drain electrodes were thermally evaporated on the semiconducting layer through a shadow mask at a rate of 0.5 Å/s. The *W* and *L* of the channels are 5600 μm and 200 μm, respectively.

P3HT Based Transistor: A solution containing methyl methacrylate (MMA), hexamethylene diacrylate (20% in molar ratio with respect to MMA), 0.01% phenylbis(2,4,6-trimethylbenzoyl)phosphine oxide and 0.07 mL toluene was drop casted on ITO-coated PEN substrate and photocured in N_2. The resulting PMMA dielectric layer was annealed at 100°C for 1 h in N_2. 50 nm P3HT was spin-coated on PMMA layer and annealed at 100°C for 1 h in N_2 to form a semiconducting layer. Finally, 50 nm Au source and drain electrodes were thermally evaporated on the semiconducting layer through a shadow mask at a rate of 0.5 Å/s. The *W* and *L* of the channels are 5600 μm and 200 μm, respectively.

For P3HT/SiO_2 based transistor, 50 nm P3HT was spin-coated on n^{++} Si wafer with 300 nm SiO_2 (with/without HMDS treatment) and annealed at 100°C for 1 h in N_2 to form a semiconducting layer. Finally, 50 nm Au source and drain electrodes were thermally evaporated on the semiconducting layer through a shadow mask at a rate of 0.5 Å/s. The *W* and *L* of the channels are 5600 μm and 200 μm, respectively.

C8-NDI/*p*-NDI blend Based Transistor: 30 nm C8-NDI/*p*-NDI was spin-coated on n^{++} Si wafer with 300 nm SiO_2 and annealed at 100°C for 1 h in N_2 to form a semiconducting layer. Finally, 50 nm Au source and drain electrodes were thermally

evaporated on the semiconducting layer through a shadow mask at a rate of 0.5 Å/s. The W and L of the channels are 5600 μm and 200 μm, respectively.

Electrical Characterization: The transistors were measured using an Everbeing CG-196 probe station connected to a Keithley SC-4200 parametric analyzer. In the I_d-V_g transfer curve measurements, the V_g sweeping rate was set as 2 V per step. All electrical characterizations were conducted in vacuum condition. All the photo-responsive characteristics of the device were evaluated by the white light with tunable light intensity from 0.07 to 4.2 mW cm^{-2}. In Figure 2c and 2d, the transistor was working in a 'sampling' mode at a constant V_g and V_d of 100 V with a holding time and interval of 0.01 s and 0.1 s, respectively. Due to the short holding time, the I_d (~ 30 nA) before light irradiation (*i.e.*, baseline in Figure 2c and 2d) is relatively low. As a result, the $I_{d,photo}/I_{d,dark}$ ratio in Figure 2c and 2d are much higher than that in Figure 2a due to their much lower I_d baselines, though the net photocurrent in Figure 2c and 2d are much smaller than that in Figure 2a.

Mobility Calculation: The values of mobility are extracted according to the following equation:

$$I^{1/2}_{\mathrm{ds,\,sat}} = (W\mu_{\mathrm{sat}}C_{\mathrm{i}} / 2L)^{1/2} (V_{\mathrm{g}} - V_{\mathrm{th}}) \quad (1)$$

Where $I_{\mathrm{ds,\,sat}}$ is the saturated drain current, W and L are the adjusted width and length of the transistor according to the crystal dimensions, respectively, C_{i} is the gate capacitance, μ_{sat} is the mobility, and V_{th} is the threshold voltage. In $I_{\mathrm{ds,\,sat}}^{1/2} - V_{\mathrm{g}}$ curves, the cross point of the tangent of the curve with the x axis (V_{g} axis) at $I_{\mathrm{ds}}^{1/2}$ equal to zero is the value of threshold voltage. The mobility can be extracted from the slope of the tangent of $I_{\mathrm{ds,\,sat}}^{1/2} - V_{\mathrm{g}}$ curves: Slope = $(W\mu_{\mathrm{sat}}C_{\mathrm{i}} / 2L)^{1/2}$.

HOMO and LUMO Calculation: The highest occupied molecular orbital (HOMO) is extracted according to the UPS measurement (Figure S8):

$$\mathrm{E_{HOMO}} = hv - (E_{cutoff} - E_{onset}) \quad (2)$$

Where the HOMO energy is determined using the incident photon energy, $hv = 21.2$ eV. E_{cutoff} is determined by linear extrapolation to zero of the yield of secondary electrons,

the left side of the Figure S8(a) and (b) displays the E_{cutoff}, and the values of C8-NDI and *p*-NDI are 16.65 eV and 16.43 eV, respectively. The panel on the Supplementary Fig. 8(a) and (b) illustrates the UPS curves concerning the valence band edges, and the E_{onset} values of C8-NDI and *p*-NDI are 1.75 eV and 1.24 eV, respectively. The lowest unoccupied molecular orbital (LUMO) levels were calculated with the optical bandgap (Eg) and equation of LUMO = HOMO + *Eg*. The respective (HOMO, LUMO) levels for C8-NDI and *p*-NDI are (–6.3, –3.15) eV and (–6.01, –2.92) eV, respectively.

3. Simulations of the Reservoir Computing

Workflow of the Reservoir in Extract Features of 2D Images: The optical images are first binarized and transferred into 32 types of 5-long optical sequences where each timestep is '1' (0.5 seconds illumination) or '0' (0.5 seconds darkness). We stimulated the *p*-NDI twelve times and observed the corresponding responses for each type of sequences. During simulation, the reservoir takes a 5-long optical sequence as input and randomly select one of the twelve corresponding responses as output feature. The resulted 140-long features are further classified with the simulated memristive organic ion gel diode crossbar arrays.

Workflow of the Reservoir in Extracting Features of 3D Videos: The event video stream is first down-sampled to 28 pixels in both spatial (x and z) directions, followed by time binning into 32 frames (*i.e.*, integrating the events in 32 equally spaced time intervals). Five evenly spaced frames from the 32 frames are taken for the downstream classification. Like the feature extraction for the 2D images, each *p*-NDI transistor pixel translates the 5-frame optical event pulse stream to a source-drain current change. The 28×28 reservoir feature map is then flattened into a 784-long vector, before being fed into the non-volatile organic readout map for classification.

Simulations of the Readout Map Training: The training of 140×18 dimensional memristive organic ion gel diode crossbar arrays was simulated. The row inputs to the memristive crossbar array are analog voltages propositional to the I_{ds} of *p*-NDI transistors in the reservoir at the final time step, while the associated currents running

through the columns of the crossbar are sampled for Softmax activation. Backpropagation was used to fit the weights of the memristive organic ion gel readout map, according to the experimentally acquired potentiation and depression characteristics (See Supplementary Fig. 24c). The categorical cross-entropy loss is minimized by the batch gradient descent (batch size = 2048) using the Adams (learning rate = 0.01) optimizer. The computed gradients are first mapped to the number of electrical pulses scaled by the standard deviation of the cycle curves and rounding to integers, before approximating the programmed diode conductance. The memristive organic ion gel diode readout layer is trained 100 epochs on each independent task with the learning rate reduced 10 times at the 70 th epoch.

The single-layered ANN and double-layered ANNs are used for comparison. The hidden layer size of the double-layered ANN is 50. Both models are optimized using Adams optimizer with learning rate as 0.01.

Software Baseline: We constructed a pure software multi-tasking reservoir computing system as a baseline for comparison. The software reservoir takes the 5-long sequence as input, considering it as a binary number, and outputs the decimal value of this 5-digit binary number, making it ideally discriminative of different 5-long sequences. The output of the reservoir is further feed into a pure software linear readout layer for classification.

4. Supporting Figures

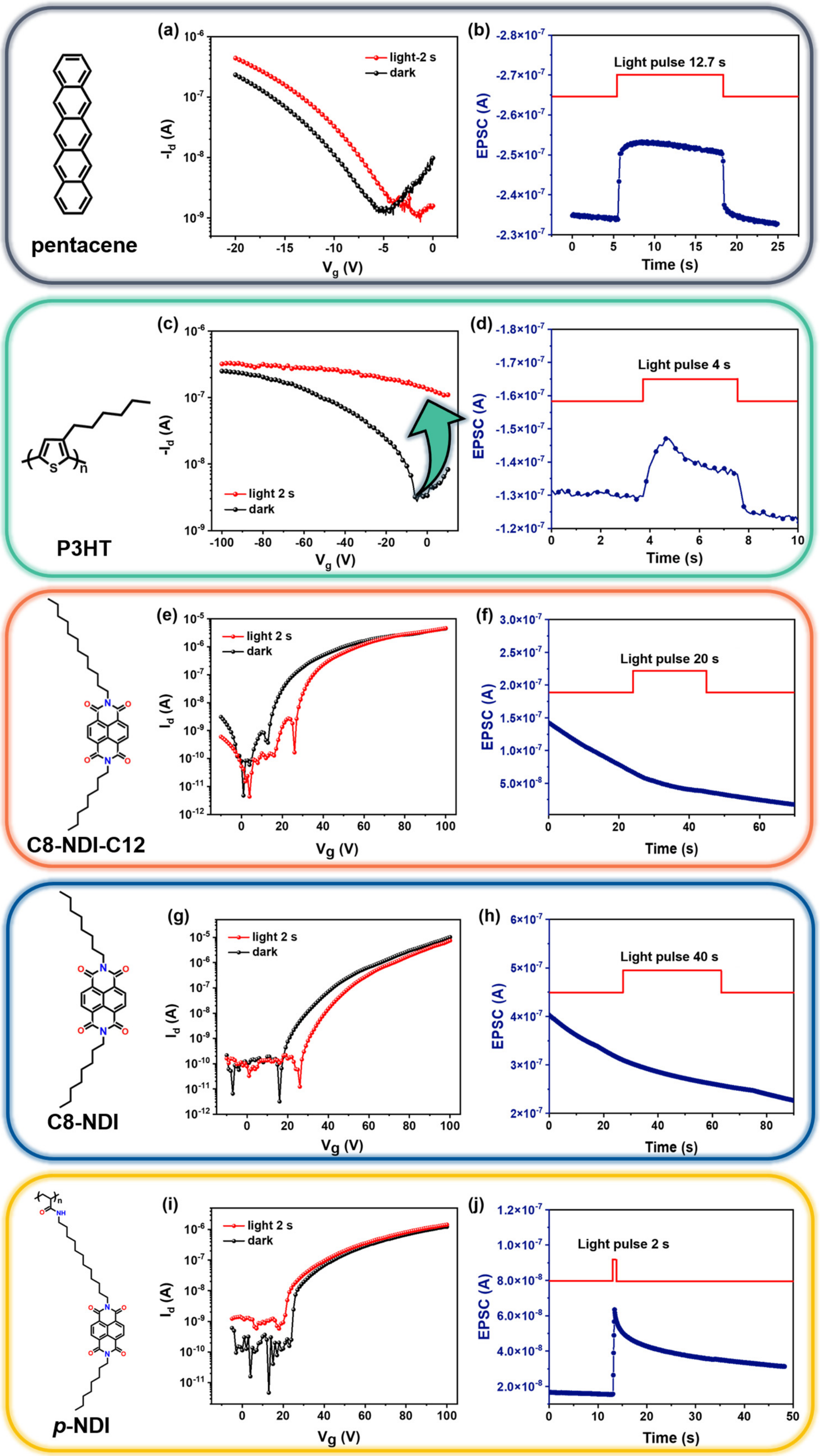

Supplementary Fig. 1 | The photocurrent responses of (a-b) pentacene, (c-d) P3HT, (e-f) C8-NDI-C12, (g-h) C8-NDI, and (i-j) *p*-NDI based transistor under a light irradiation of 4.2 mW cm^{-2}. The dielectric layer is SiO_2 for all devices.

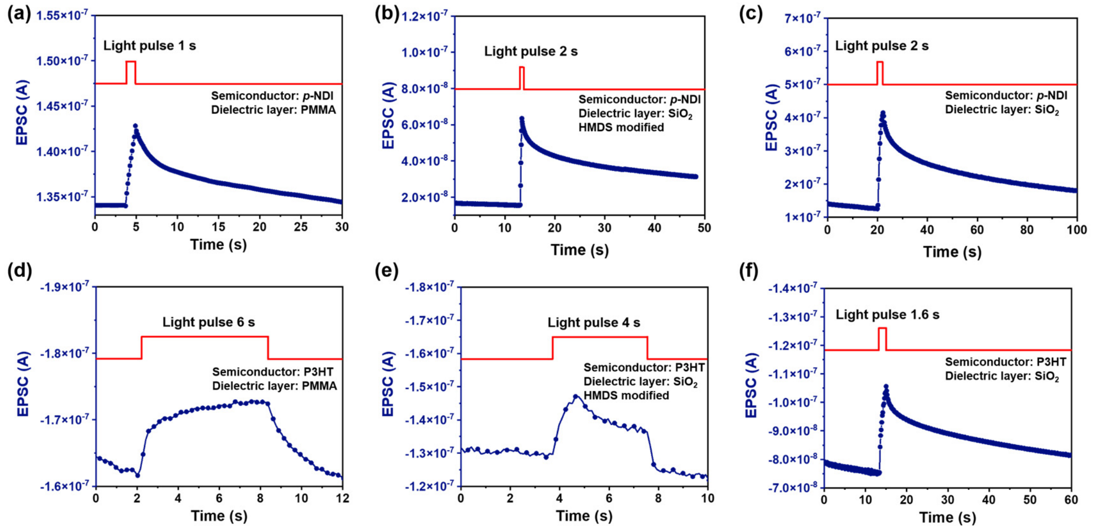

Supplementary Fig. 2 | Photocurrent response of *p*-NDI transistors with (a) PMMA and (b) HMDS modified SiO2, and (c) SiO2 as dielectric layer. Photocurrent response of P3HT transistors with (d) PMMA and (e) HMDS modified SiO2, and (f) SiO2 as dielectric layer. HMDS: Hexamethyldisilane. Light intensity: 4.2 mW cm^{-2}. The transistors were working in a 'sampling' mode at a constant V_g and V_d of 100 V or −100V with a holding time and interval of 0.01 s and 0.1 s, respectively.

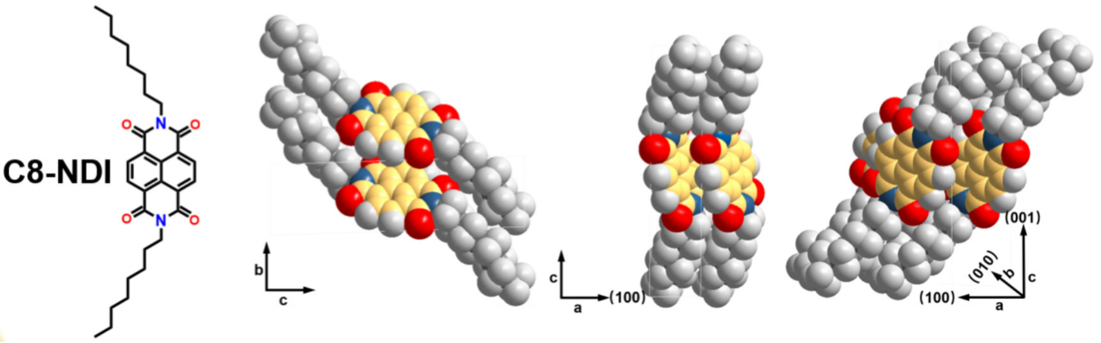

Supplementary Fig. 3 | The chemical structure and the crystal structure of C8-NDI. Triclinic P-1, a = 4.7814(2), b = 6.5335(3), c = 22.7590(9) Å, α= 87.874(2), β = 88.991(5), γ = 75.754(3) °, V = 688.91(5) Å3, Z = 1.[1]

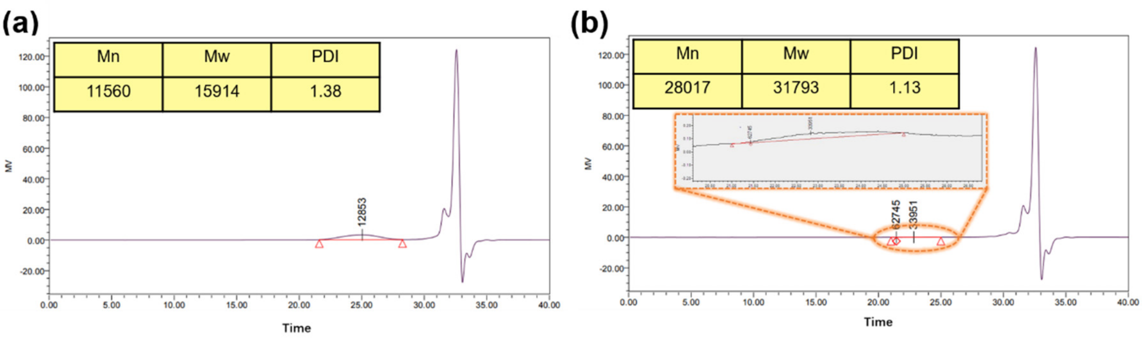

Supplementary Fig. 4 | GPC measurement of (a) poly (pentafluorophenyl acrylate) (pPFPA), (b) *p*-NDI.

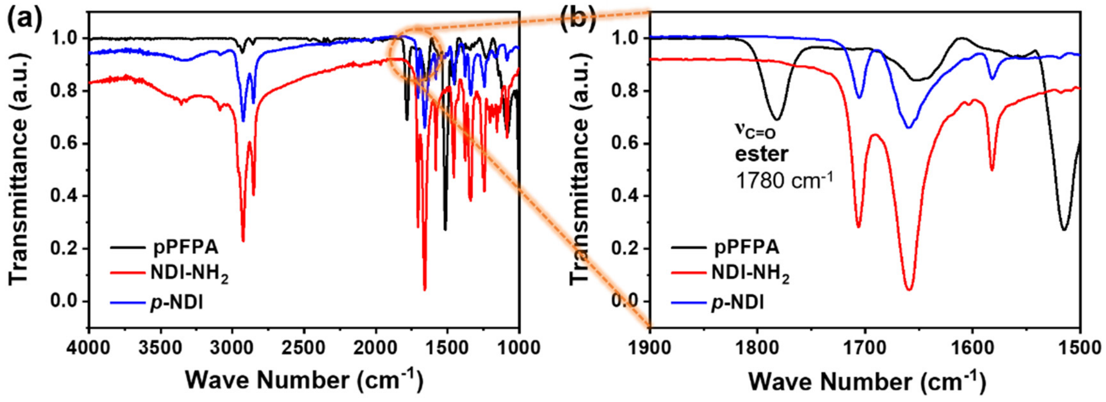

Supplementary Fig. 5 | (a) ATR spectrum of pPFPA (black curve), *p*-NDI (blue curve) and NDI-NH_2 (red curve). (b) Enlarged spectrum with detailed peak assignments with wavenumber from 1500 to 1900 cm^{-1}.

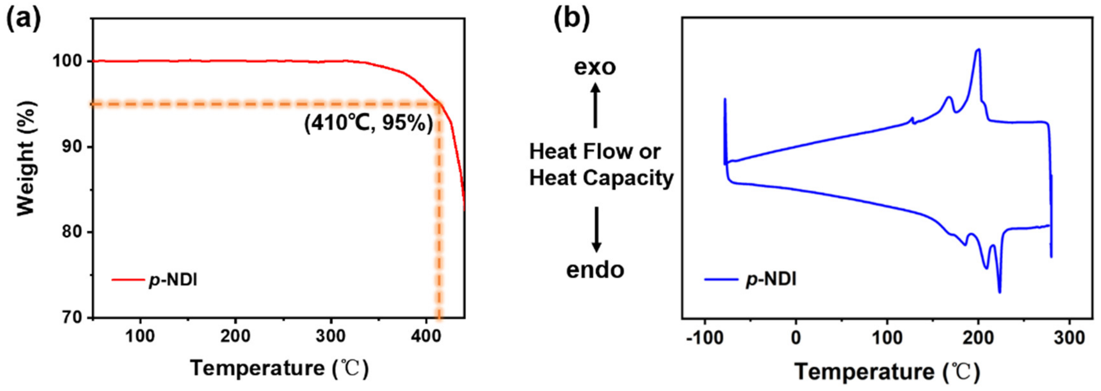

Supplementary Fig. 6 | (a) Thermogravimetric Analysis (TGA) curve of *p*-NDI film. (b) Differential scanning calorimetry (DSC) characterization of *p*-NDI film.

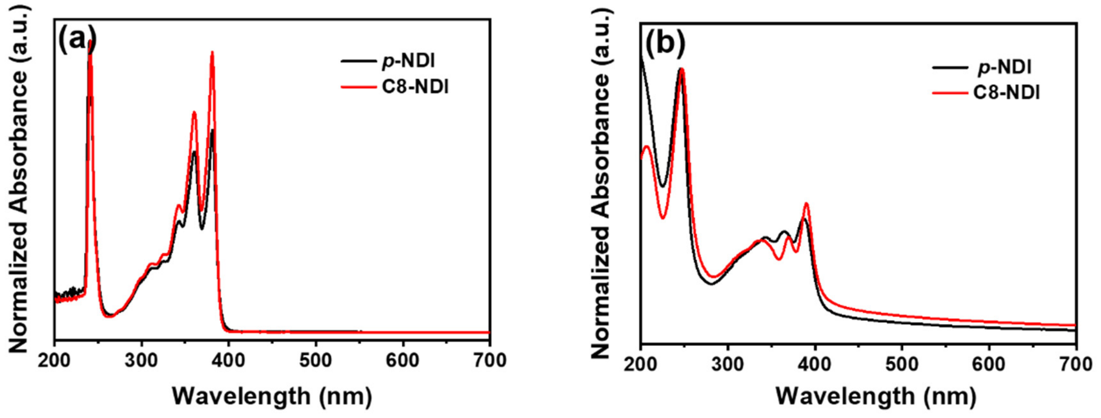

Supplementary Fig. 7 | UV-vis absorption spectra of (a) the *p*-NDI and C8-NDI solution, and (b) the *p*-NDI and C8-NDI film.

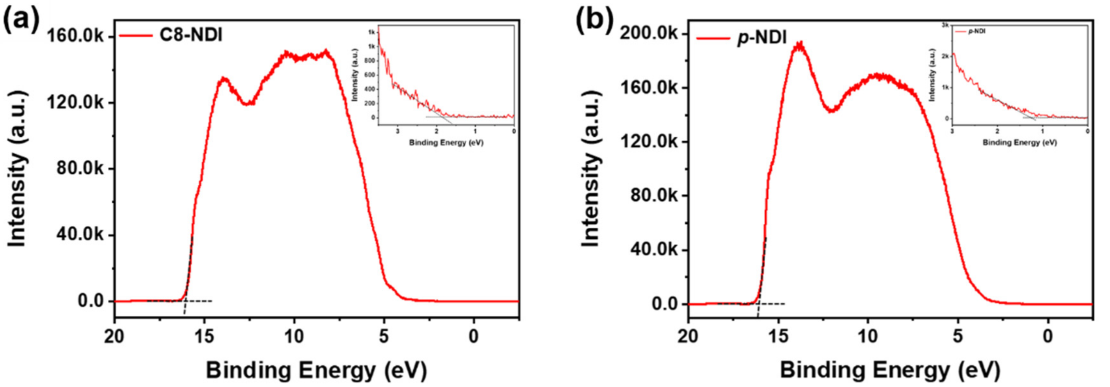

Supplementary Fig. 8 | UPS characterizations of (a) C8-NDI, and (b) *p*-NDI film.

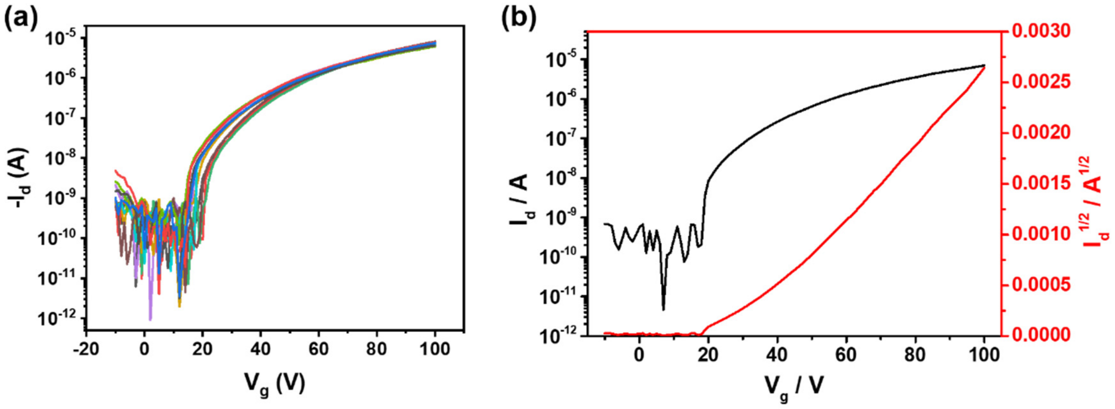

Supplementary Fig. 9 | (a) Stability of transfer curves by repeated sweeping tests, and (b) typical n-type transfer curve of the *p*-NDI based transistor. V_g sweeps from −10 to 100 V, V_d holds at 100 V.

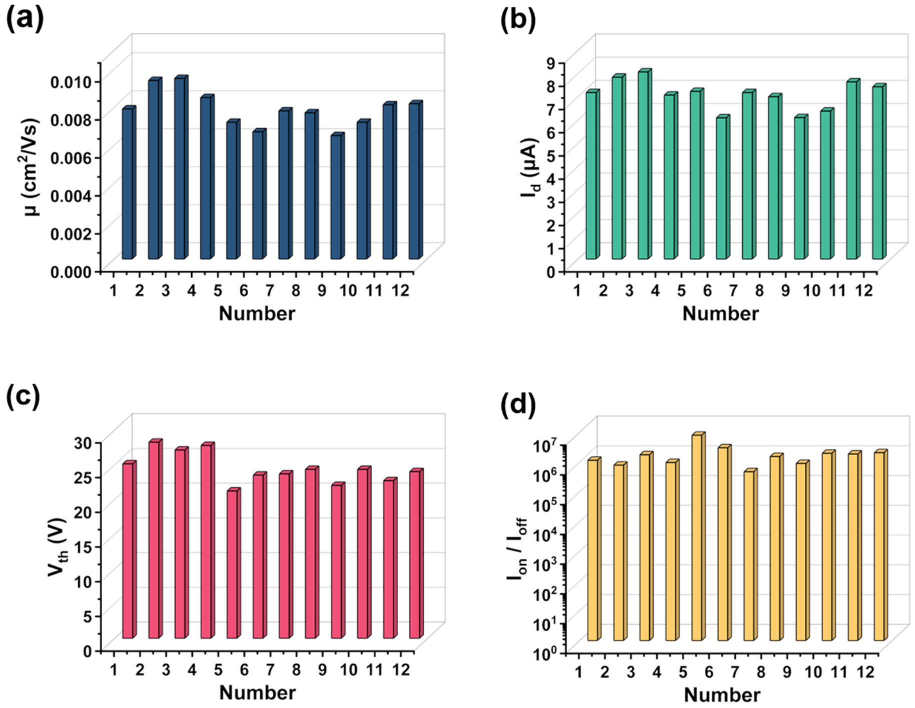

Supplementary Fig. 10 | Statistic of (a) μ, (b) I_d, (c) V_{th}, (d) on/off ratio of the *p*-NDI based transistor under dark.

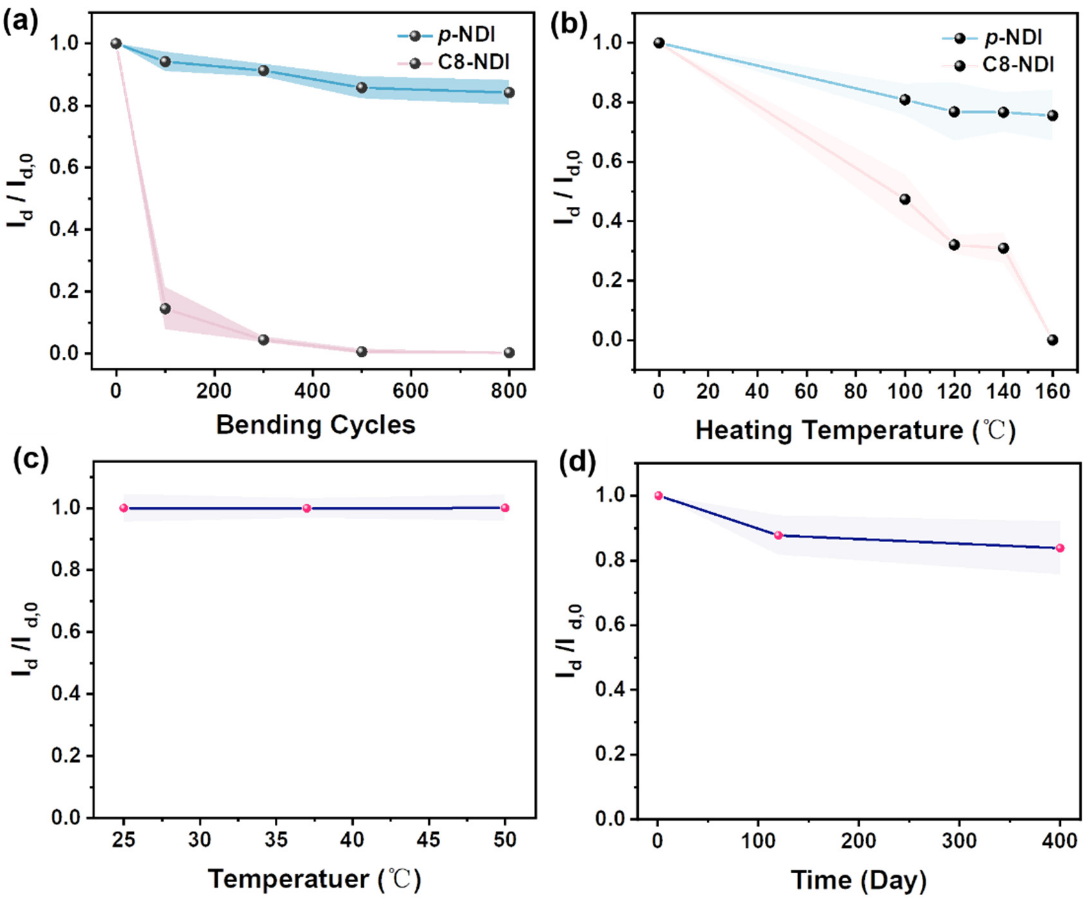

Supplementary Fig. 11 | (a) I_d change over bending cycles under a bending radius of 5.5 mm. (b) I_d change after different heating temperatures. (c) I_d change of *p*-NDI-based transistors at different test temperatures. (d) The I_d change of *p*-NDI-based transistors over storage time. Each data point is an average of five repeated tests. All statistical data are presented as a mean ± SD (n = 5).

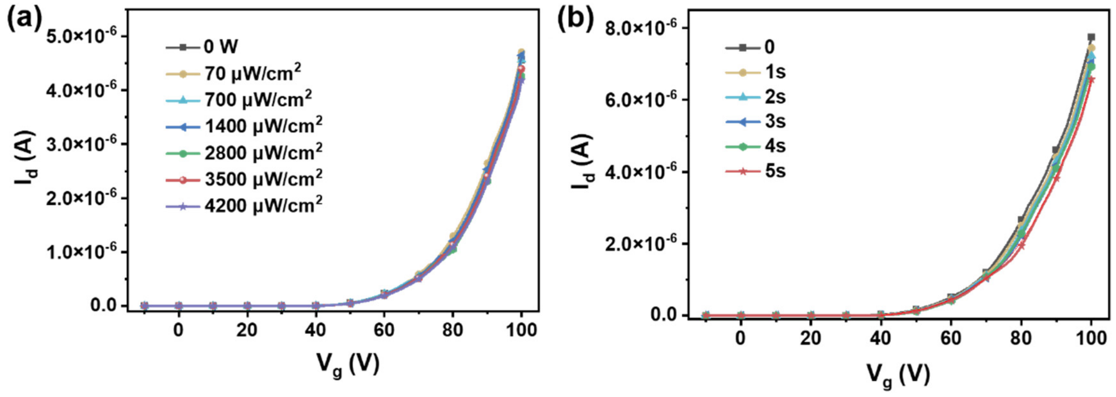

Supplementary Fig. 12 | (a) The transfer curves of C8-NDI based transistor under different light intensities for 5 s, (b) and under 4.2 mW cm^{-2} light intensities with different pulse widths. V_g sweeps from −10 to 100 V, V_d holds at 100 V.

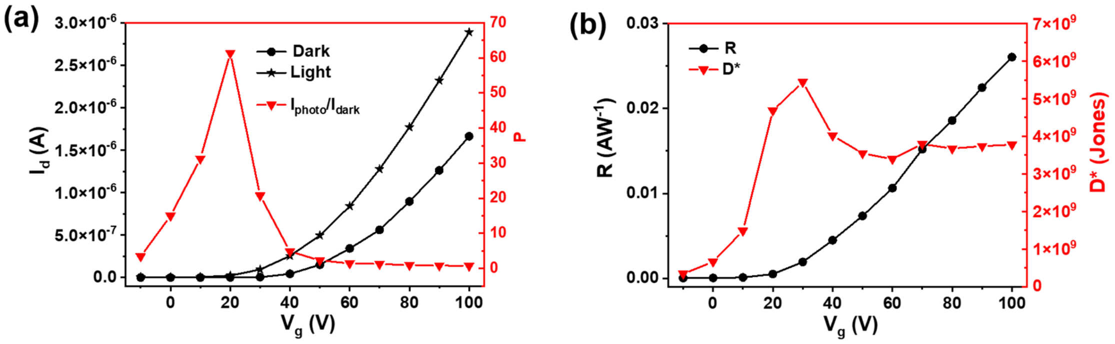

Supplementary Fig. 13 | (a) Typical transfer characteristics of *p*-NDI-based transistors in the dark and under a light intensity of 4.2 mW cm^{-2} for 5 s, and the *P* as a function of V_g. (b) The *R* and *D** as a function of V_g. V_d holds at 100 V.

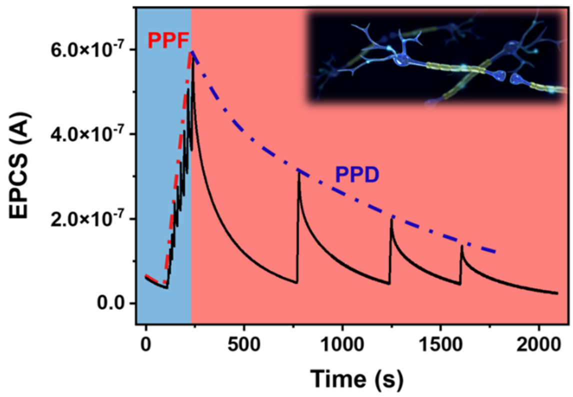

Supplementary Fig. 14 | Typical synaptic behaviors of *p*-NDI transistors. PPF: paired pulse facilitation, PPD: paired pulse depression, SRDP: spike-rate dependent plasticity. The transistor was working in a 'sampling' mode at a constant V_g and V_d of 100 V with a holding time and interval of 0.01 s and 0.1 s, respectively. The light intensity and pulse width for each spike is 1.4 mW cm^{-2} and 5 s, respectively.

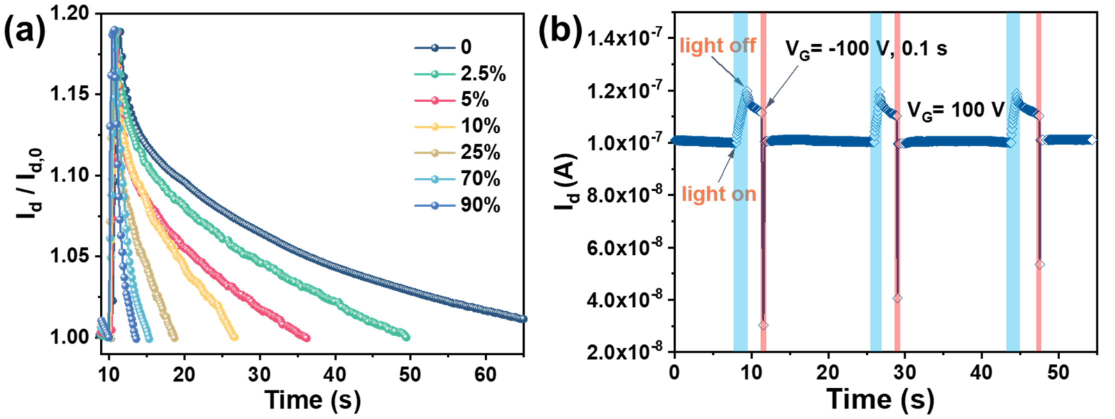

Supplementary Fig. 15 | (a) The photocurrent decay curves of C8-NDI/*p*-NDI blend semiconducting layer-based transistors at respective C8-NDI weight percentage of 0%, 2.5%, 5%, 10%, 25%, 70%, and 90%. (b) The recovery of photocurrent after reset by an electrical pulse (V_g = –100 V, 0.1 s).

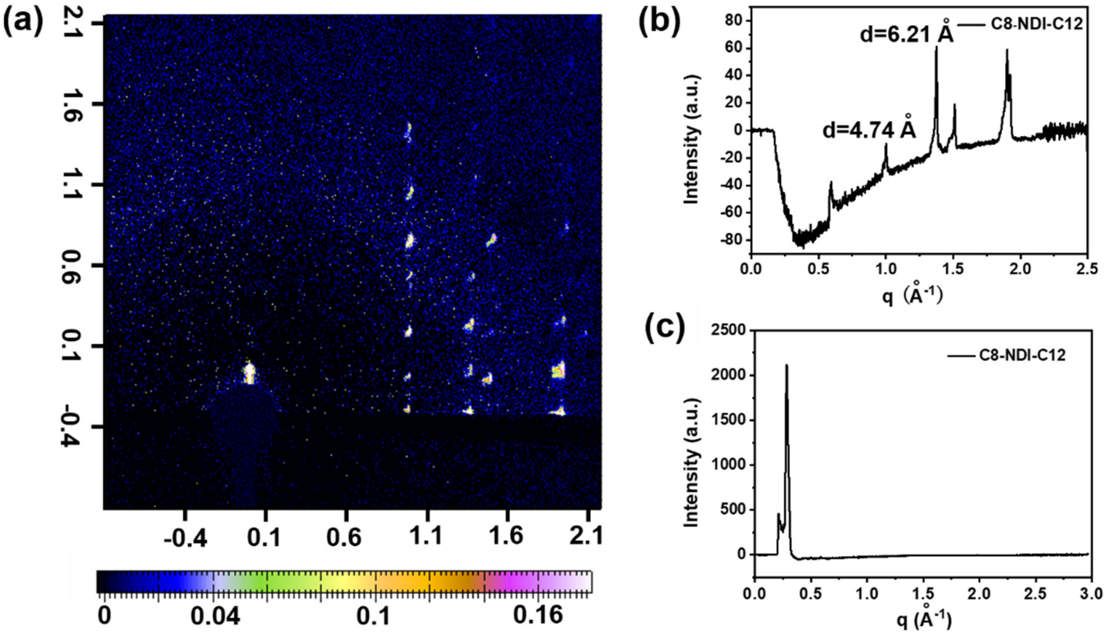

Supplementary Fig. 16 | (a) 2D grazing-incidence X-ray diffraction (GIXD) of C8-NDI-C12 films, with the in-plane diffraction peaks shown in (b), and out-of-plane diffraction peaks shown in (c).

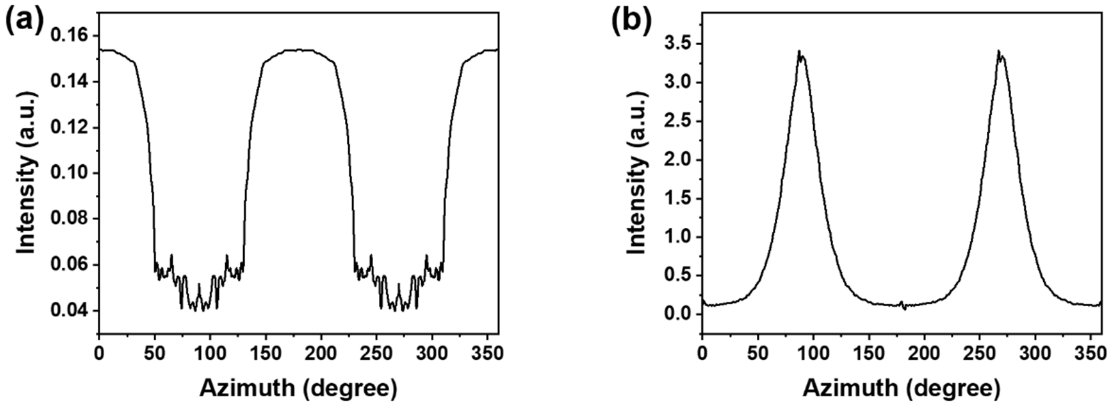

Supplementary Fig. 17 | Azimuthal intensity profiles of (a) q4 and (b) q1 from Fig. 3e.

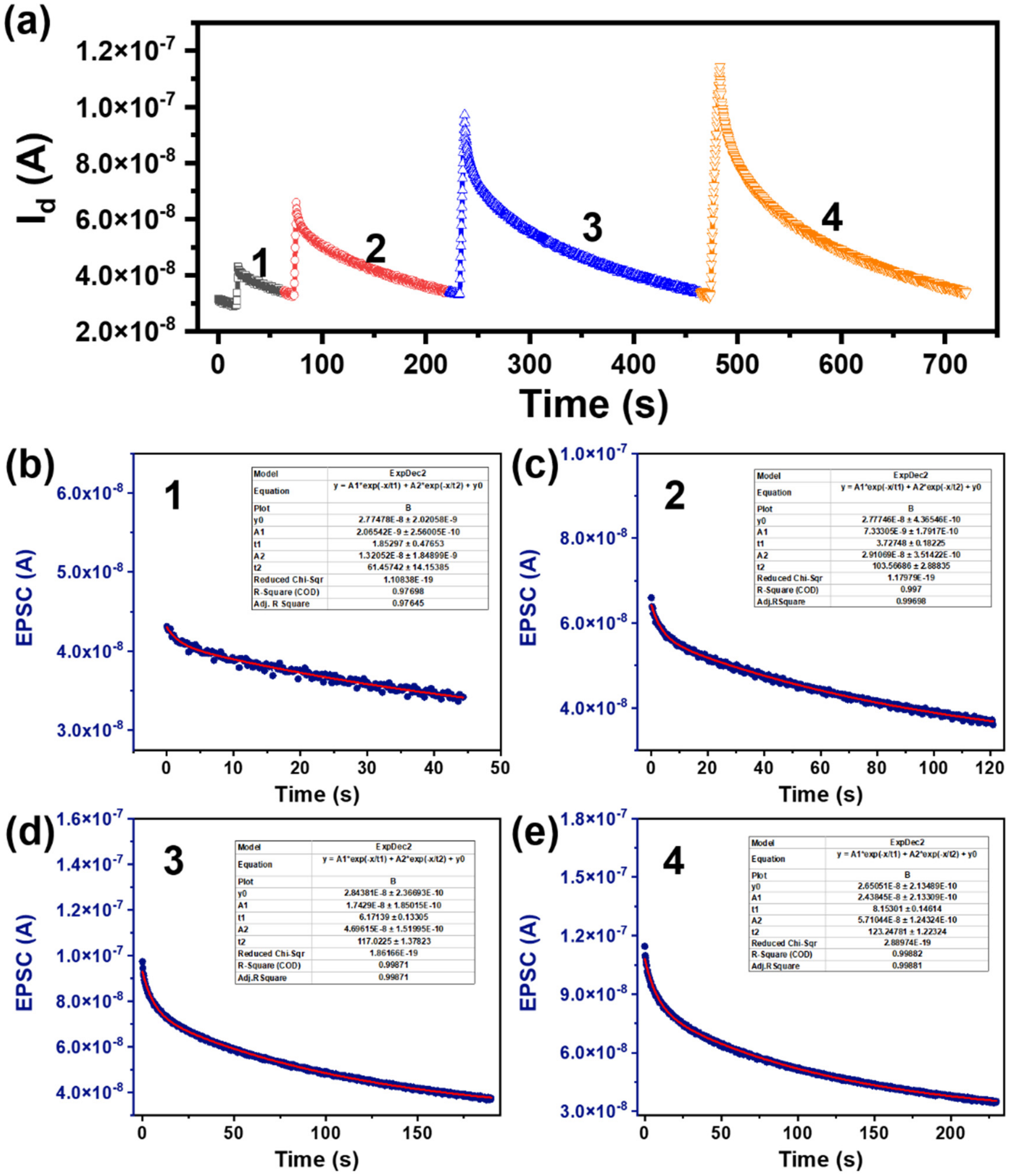

Supplementary Fig. 18 | Fitting the photocurrent decay curves with a double exponential function: $I_d = y_0 + A_1 \cdot \exp(-\Delta T/\tau_1) + A_2 \cdot \exp(-\Delta T/\tau_2)$. (a) The photocurrent response of *p*-NDI-based transistor under 700 μW cm^{-2} light irradiation with different pulse widths shown in Figure 2c. (b-d) The fitting of four corresponding photocurrent decay curves marked with 1, 2, 3, and 4 in (a).

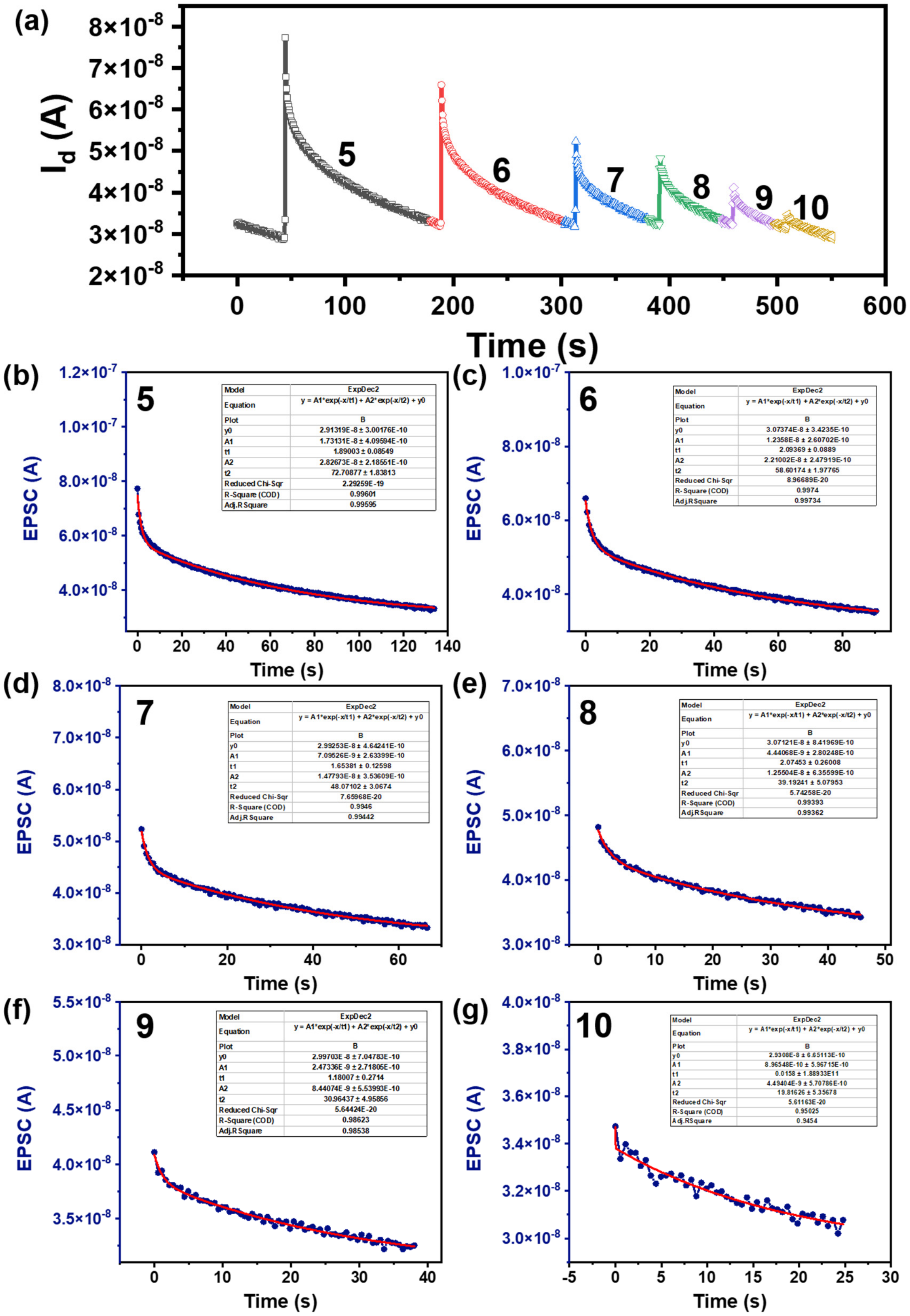

Supplementary Fig. 19 | Fitting the photocurrent decay curves with a double exponential function: $I_d = y_0 + A_1 \cdot \exp(-\Delta T/\tau_1) + A_2 \cdot \exp(-\Delta T/\tau_2)$. (a) The photocurrent response of *p*-NDI-based transistor under 1 s light irradiation with different intensities shown in Figure 2d. (b-g) The fitting of six corresponding photocurrent decay curves marked with 5, 6, 7, 8, 9, and 10 in (a).

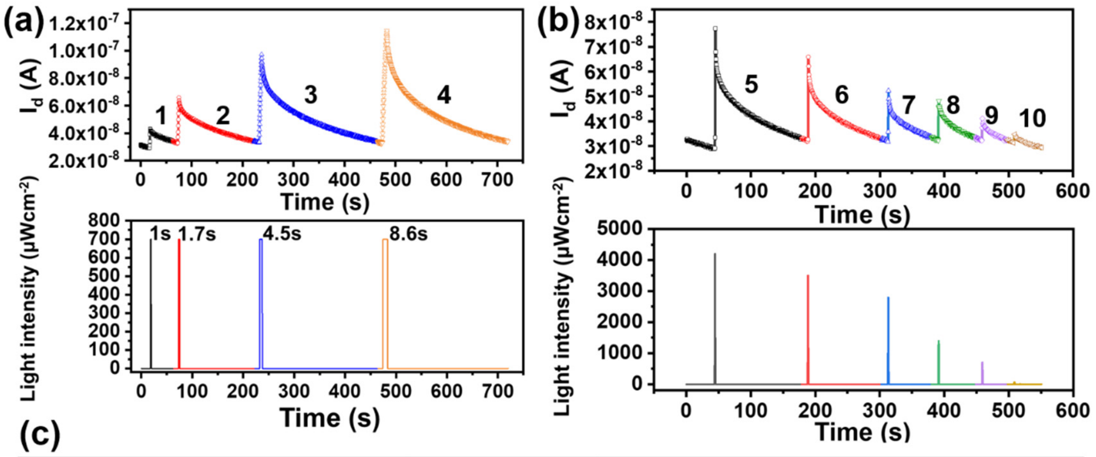

(c)

	1	2	3	4	5	6	7	8	9	10
τ_1 (s)	1.85	3.73	6.77	8.15	1.89	2.09	1.65	2.07	1.18	0.016
τ_2 (s)	61.46	103.57	127.82	123.25	72.71	58.6	48.07	39.19	30.96	19.82
τ_{avg} (s)	53.4	83.48	87.04	88.79	45.84	38.39	33.02	29.52	19.82	16.52

Supplementary Fig. 20 | The photocurrent decay curves of *p*-NDI-based transistor after being irradiated by (a) 700 μW cm^{-2} light with different pulse widths; (b) different light pulse intensities with a pulse width of 1 s. (c) The resulting decay time constant τ_1 and τ_2 by fitting the photocurrent decay curves with a double exponential function: $I_d = y_0 + A_1 \bullet \exp(-\Delta T/\tau_1) + A_2 \bullet \exp(-\Delta T/\tau_2)$, where A_1 and A_2 represent the initial rapid and slow phase facilitation magnitudes, respectively. τ_1 ranges from 0.8 to 4.61 s, whereas τ_2 ranges from 35 to 452 s, corresponding to the coexistence of both shallow level and deep level trapping states.

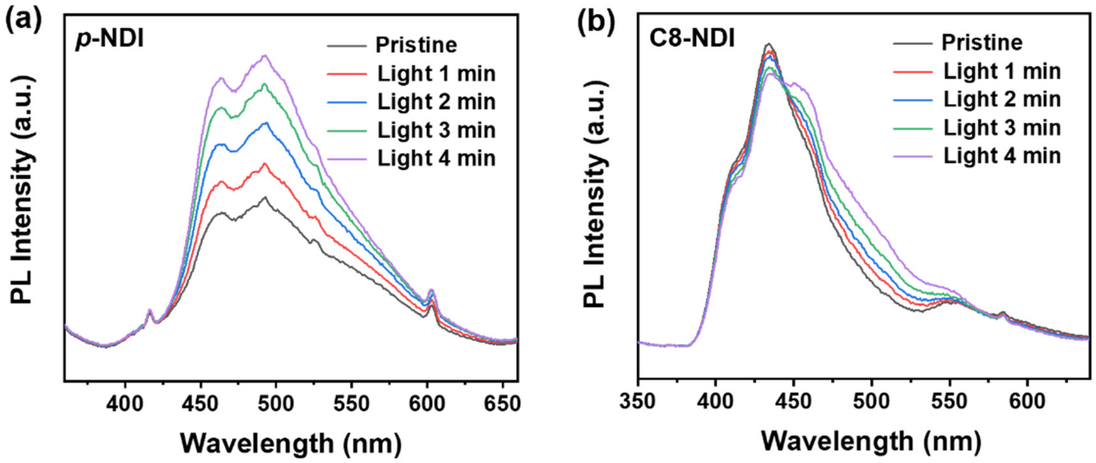

Supplementary Fig. 21 | The PL emission evolution of (a) a *p*-NDI film, and (b) a C8-NDI film over light irradiation time. (Excitation wavelength: 365 nm, light intensity: 2 W cm^{-2})

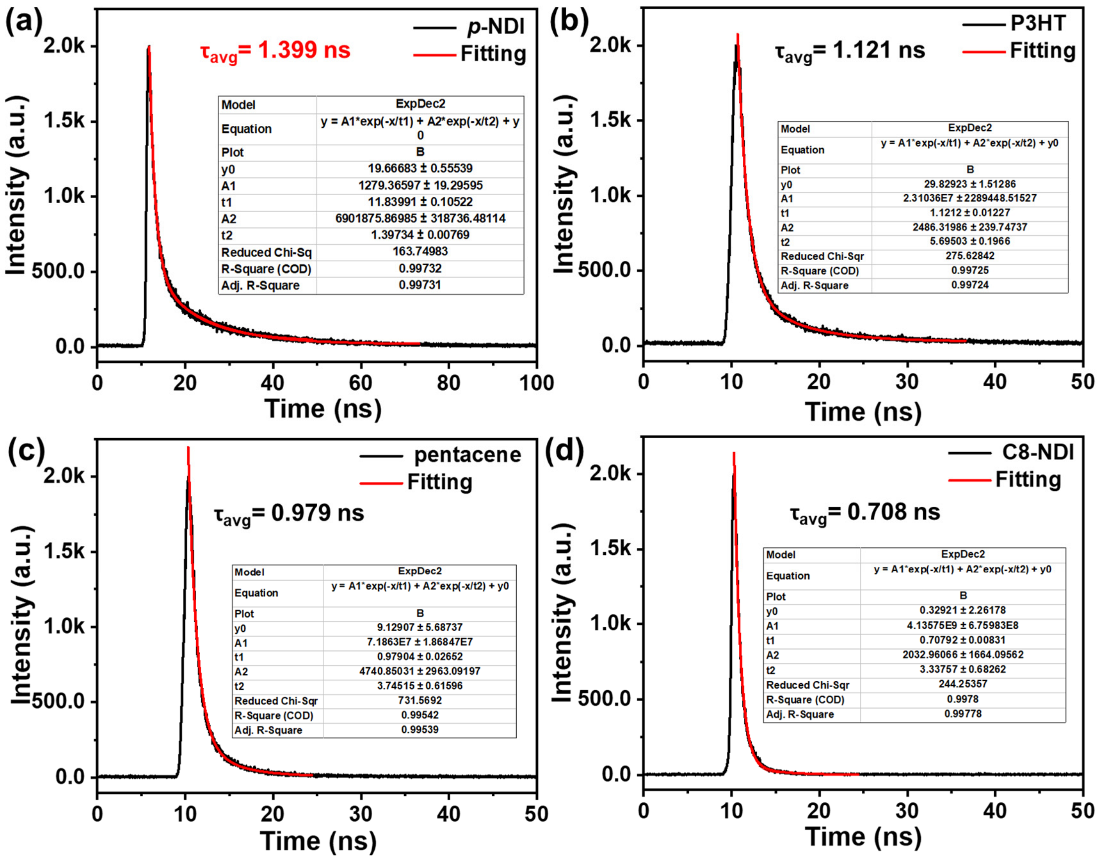

Supplementary Fig. 22 | Time-resolved photoluminescence (TR-PL) analysis of (a) *p*-NDI film (λ_{ex}: 501 nm), (b) P3HT film (λ_{ex}: 490 nm), (c) pentacene film (λ_{ex}: 550 nm), and (d) C8-NDI film (λ_{ex}: 440 nm).

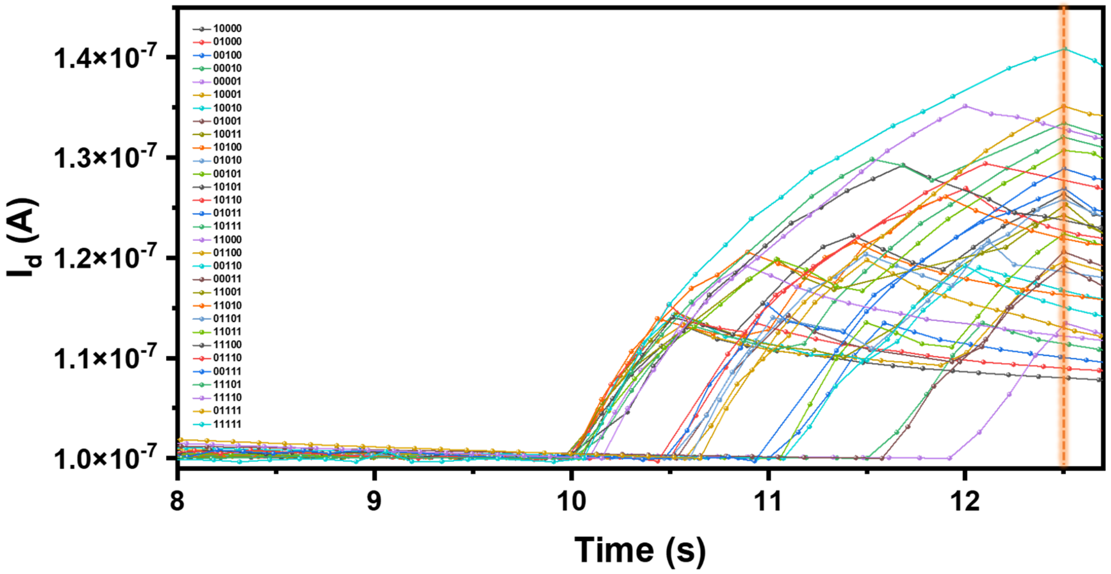

Supplementary Fig. 23 | Photo-response to thirty-one different pulse streams of *p*-NDI transistor-based RC. (The pulse stream 00000 is the baseline of the curves, light pulse frequency: 2 Hz)

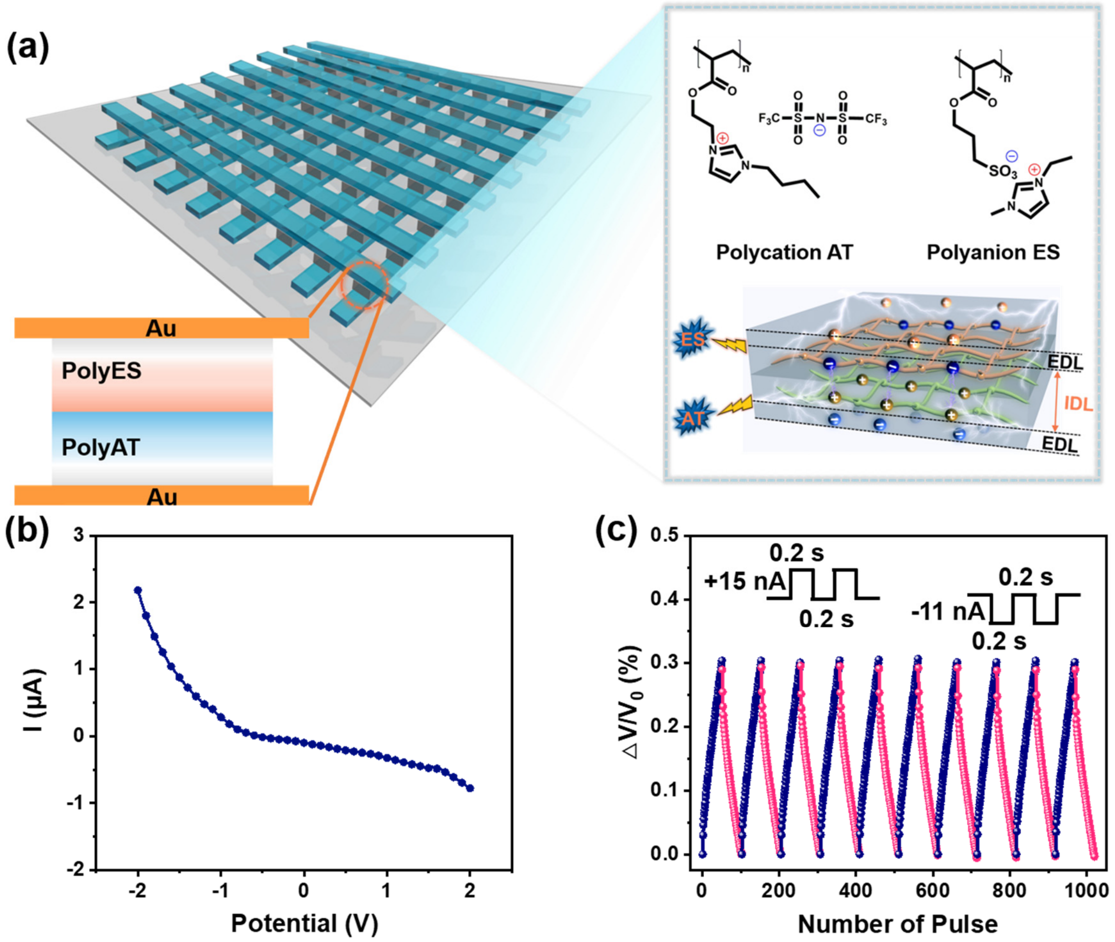

Supplementary Fig. 24 | (a) Schematic illustration of the organic ion gel diode array, and chemical structures of the polyanion ES and polycation AT. (b) *I*−*V* curves, and (c) the potentiation and depression cycles of the organic ion gel diode.

The structure of the memristive devices: a polyAT film is casted on the Au electrode. Sequentially, a layer of polyES is casted on the polyAT film, resulting to a bilayered polymeric heterojunction with an ionic double layer (IDL), much like the depletion layer at a *p-n* semiconductor junction. Finally, another Au electrode is deposited on the polyES film, giving rise to a memristive device with two electrical double layers (EDL) at the respective Au/polyAT and Au/polyES interfaces. Electrical characterization of the memristive devices reveal a typical diode behavior (Figure S24b).

The organic memristive diodes are used as programmable weights of the readout map (a fully connected feed forward layer). The conductance of the diodes, in the form of a conductance matrix, is multiplied with the features (in the form of voltage vector) generated by the *p*-NDI reservoir. The current through each diode is thus the multiplication of its input voltage and conductance, governed by the Ohm's law. The current of each output node sums up the current of individual diode thanks to Kirchoff's current law, which computes the weighted summation (or multiplication between the conductance matrix and input voltage vector) for subsequent classification.

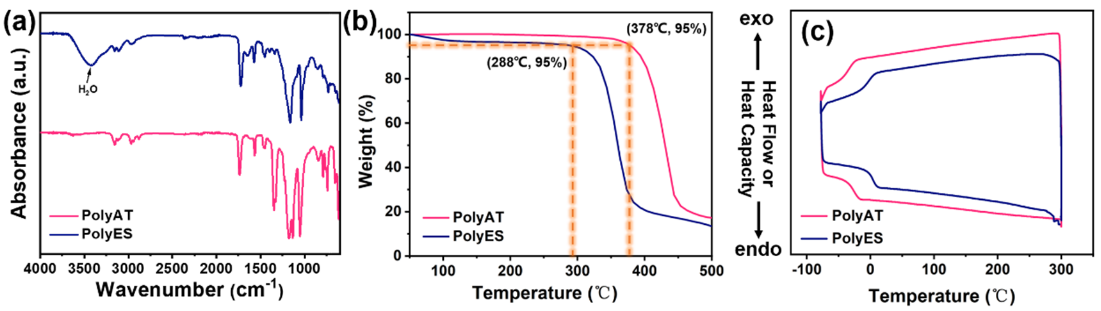

Supplementary Fig. 25 | (a) ATR characterization, (b) thermogravimetric analysis, and (c) differential scanning calorimetry measurements of polyAT and polyES gels.

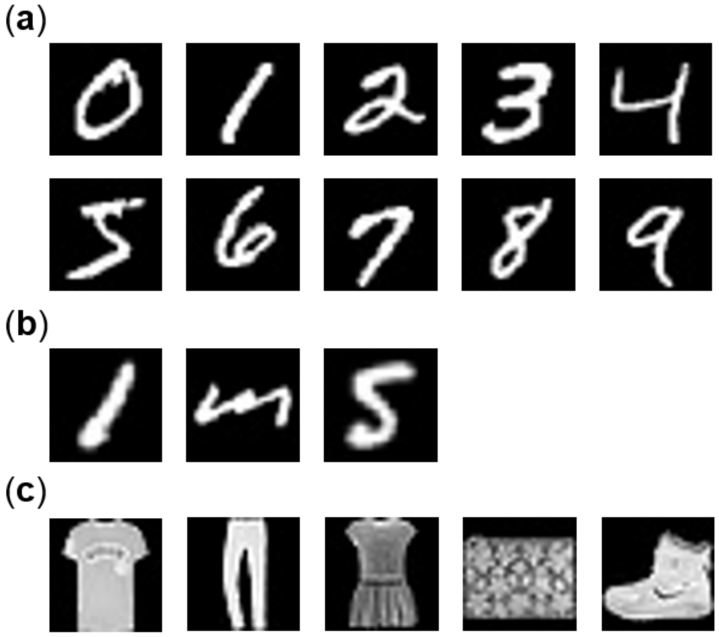

Supplementary Fig. 26 | Datasets for the multi-task computing. (a) Digital size dataset, using the MNIST dataset. (b) Letter-size dataset, using three classes ('L', 'M', and 'S') from the Extended-MNIST dataset. (c) the garment dataset, using five classes from the Fashion-MNIST dataset.

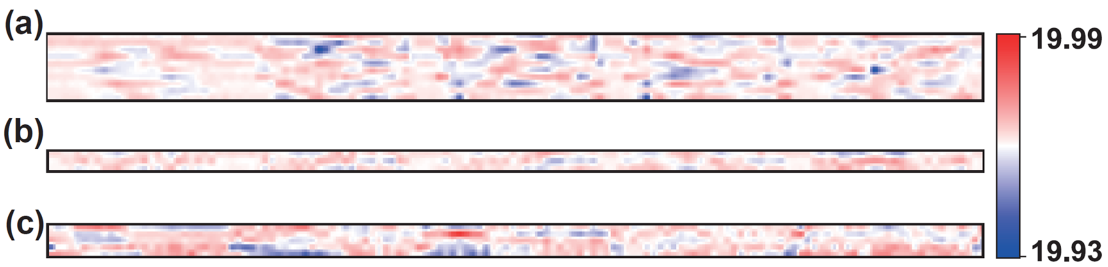

Supplementary Fig. 27 | Final readout layer weight map for classifying the digits (a), the letters (b), and the garments (c).

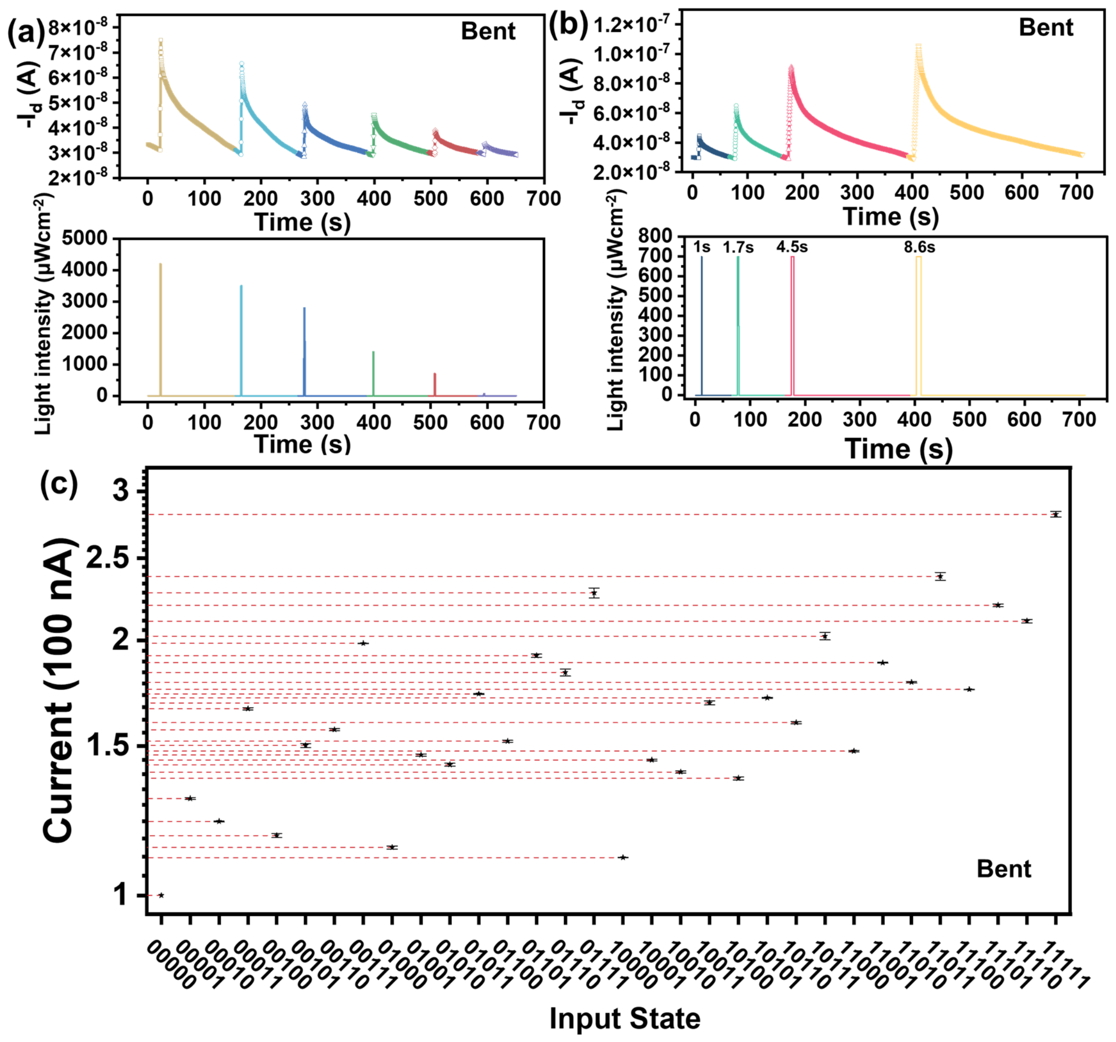

Supplementary Fig. 28 | The photocurrent response of *p*-NDI-based transistor at bending state (bending radius: 5 mm) under (a) 1 s light irradiation with different intensities and (b) 700 μW cm^{-2} light irradiation with different pulse widths. The transistor was working in a 'sampling' mode at a constant V_g and V_d of 100 V with a holding time and interval of 0.01 s and 0.1 s, respectively. (c) Experimental read-current responses generated by thirty-two optical pulse train inputs ranging from (00000) to (11111). The optical pulse is applied to the transistor every 2 s. Each data point is the average of twelve repeated tests. All statistical data are presented as a mean ± SD (n = 12).

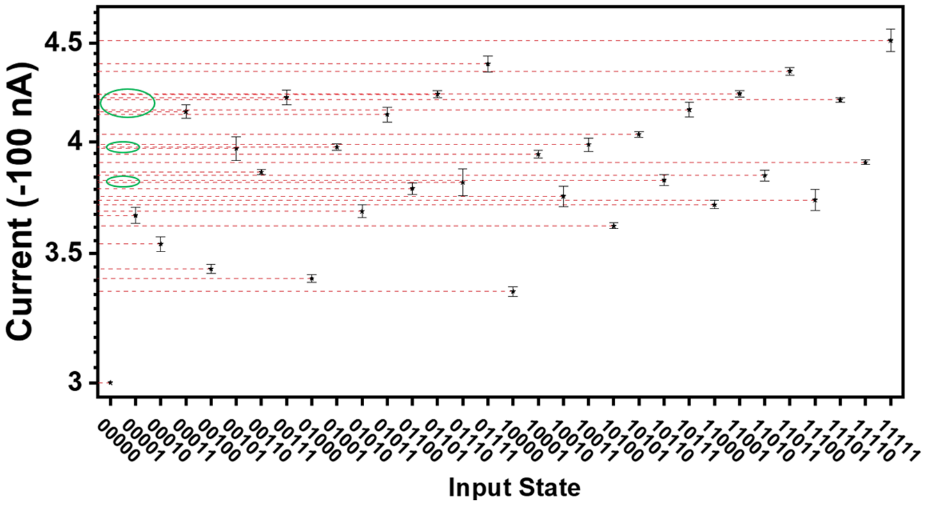

Supplementary Fig. 29 | Experimental read-current responses of a P3HT/SiO_2 transistor generated by thirty-two optical pulse train inputs ranging from (00000) to (11111). The green circles indicate the poorly separated photocurrent responses. Each data point is the average of twelve repeated tests.

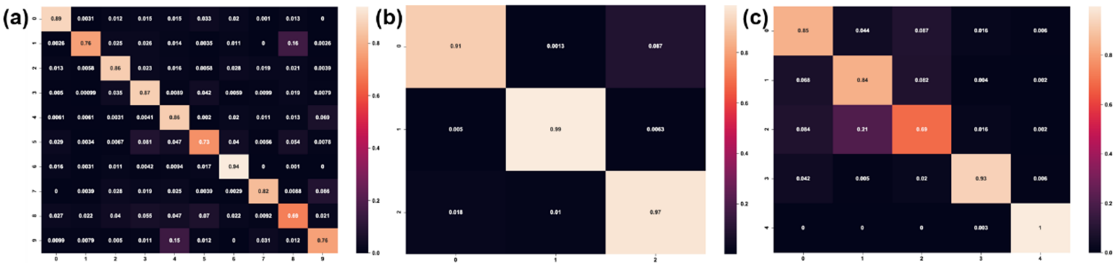

Supplementary Fig. 30 | Confusion matrices of the P3HT/SiO_2 reservoir computing system on recognizing: (a) numbers, (b) handwritten letters, and (c) classifies a variety of costumes.

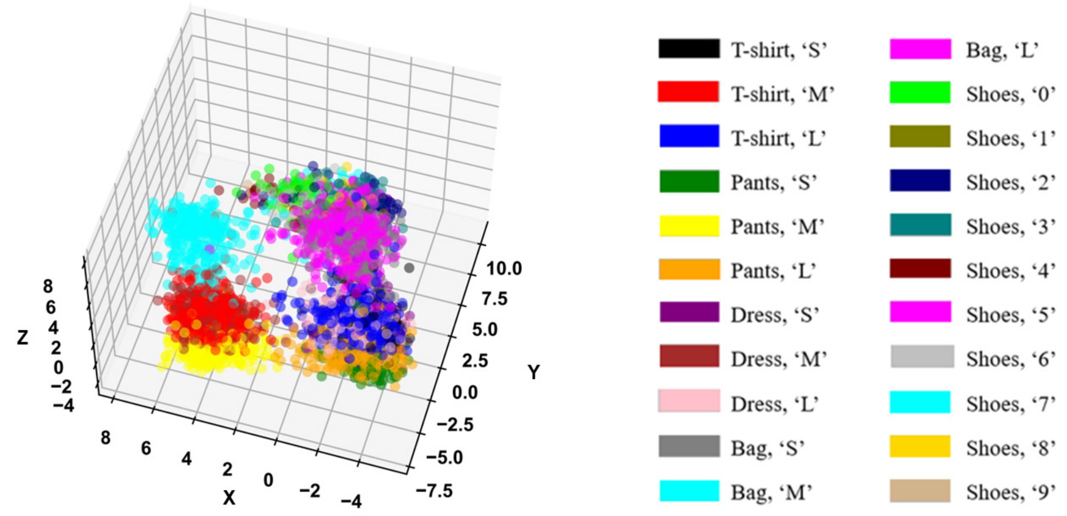

Supplementary Fig. 31 | The 3D visualization of the distribution of feature vectors of all classes, produced by the *p*-NDI in-sensor reservoir, using linear discriminative analysis (LDA) for dimensionality reduction.

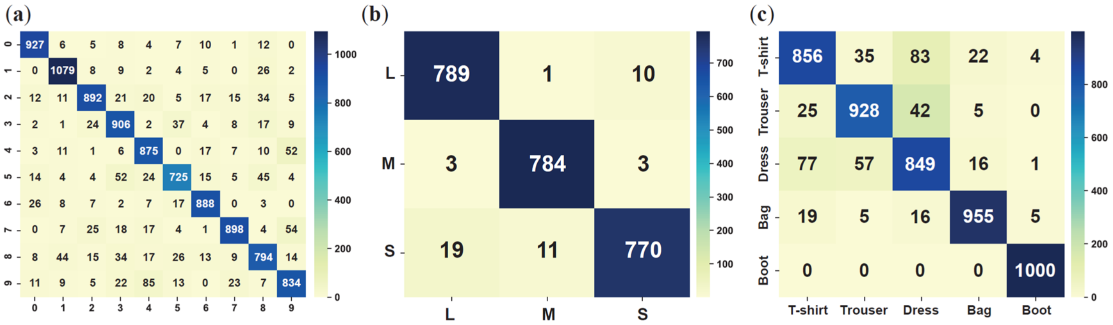

Supplementary Fig. 32 | Non-normalized confusion matrices of the digits (a), letters (b), and garments (c) for the *p*-NDI in-sensor reservoir.

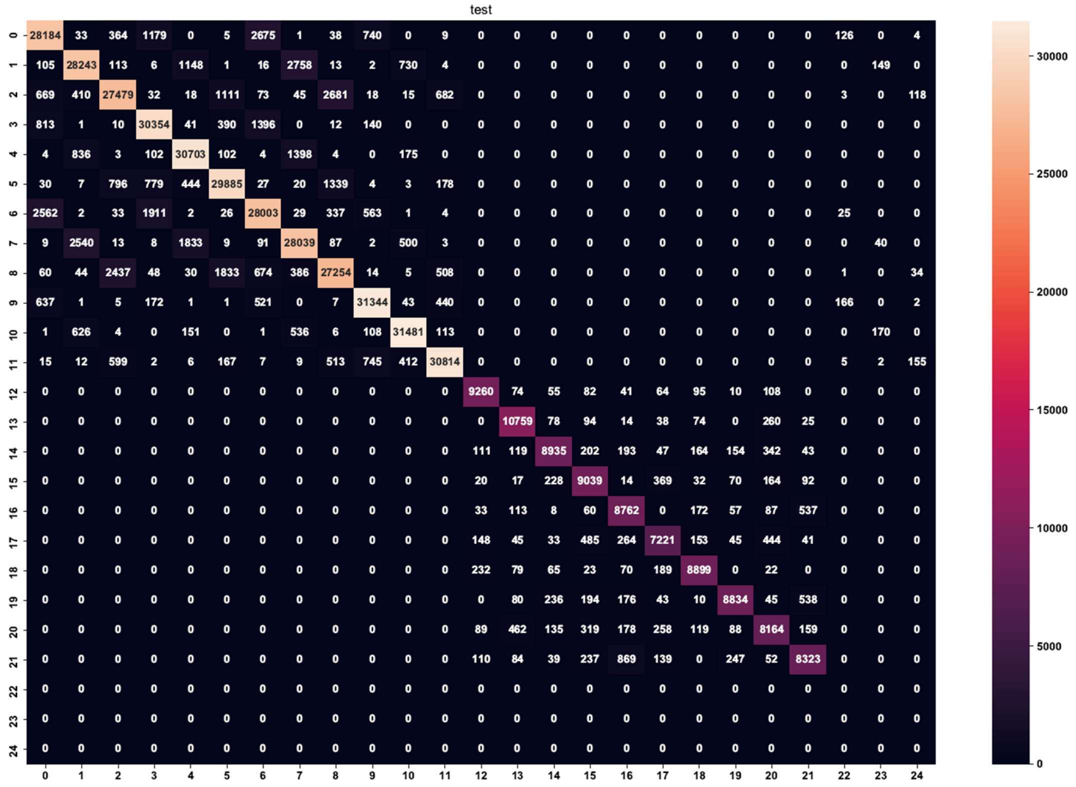

Supplementary Fig. 33 | Overall confusion matrix of the *p*-NDI in-sensor reservoir for the multi-task learning.

Supplementary Table 1 | The independent and overall accuracies of *p*-NDI based-RC in recognizing the letters, numbers, and the garments at different optical pulse input frequencies.

Frequency	Letter	Number	Garment	Overall
0.5 Hz	97.58%	90.63%	89.34%	87.67%
0.5 Hz (Bent)	97.25%	85.37%	88.48%	83.67%
2 Hz	98.04%	88.18%	91.76%	88.00%
0.5 Hz (P3HT)	95.75%	81.75%	86.08%	77.86%

5. Synthesis of Materials

5.1 Synthesis of Semiconducting Polymer (*p*-NDI)

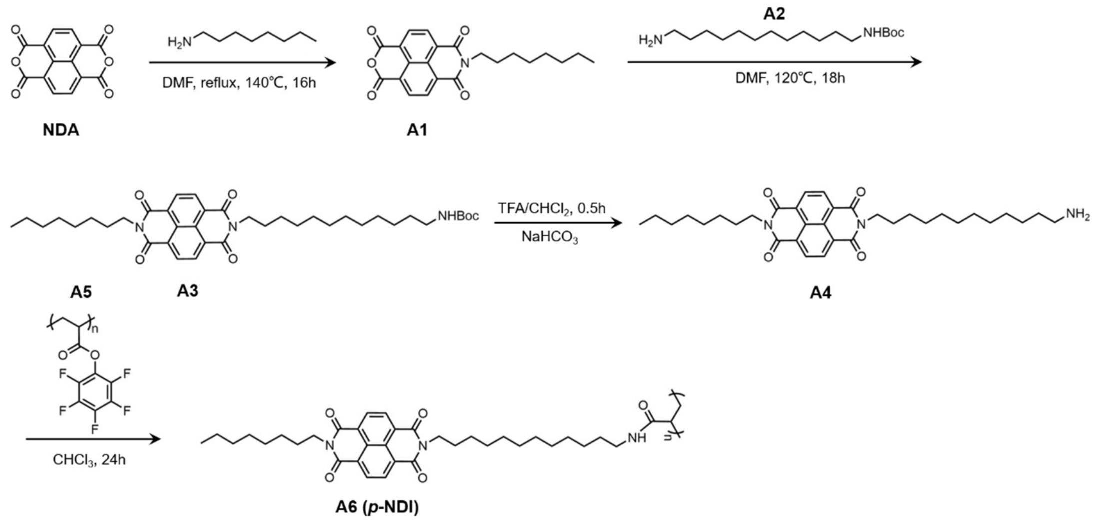

Supplementary Scheme 1. Synthesis process of semiconducting polymer (***p*-NDI**).

Compound **A1**. 1,4,5,8-Naphthalenetetracarboxylic dianhydride (1 g, 3.73 mmol) was added to a dry 100 mL flash. To the solid was added dry DMF (20 mL) and heated to 140°C under stirring in nitrogen atmosphere. After stirring for 10 min, the N-octylamine (0.385 g, 2.98 mmol) soluble in 5 ml dry DMF, and then was added dropwise. Cooling the reaction mixture in a refrigerator for 2 h. DMF was removed under reduced pressure and heated to 80°C. Added 50 ml of acetone to dissolve it, cooled in a refrigerator overnight and the resulting precipitate was filtered. The filtrate was evaporated and the residue was purified by silica gel column with DCM. Yield, 31.6% (0.446 g). ^{1}H NMR (400 MHz, $CDCl_3$) δ: 8.82 (s, 4H), 4.24-4.17 (m, 2H), 1.74 (dd, J = 15.4, 7.9 Hz, 2H), 1.46-1.24 (m, 10H), 0.88 (t, J = 6.8 Hz, 3H).

Compound **A2**. According to the reported literatures,[2] 1, 12-Dodecanediamine (1 g, 5 mmol) was added to a dry 100 mL flash and dissolved in $CHCl_3$ (20mL). After stirring for 10 min, the di-tert-butyl dicarbonate (Boc_2O) (0.218 g, 1 mmol) dissolved in $CHCl_3$ (50 mL), and then added dropwise over 3 h at 0°C. After stirring overnight, dichloromethane was added and the solution was washed water and the mixture was dried over anhydrous Na_2SO_4 and concentrated in vacuum. The residue was purified by silica gel column with a solution of 1% Et_3N/10% MeOH in $CHCl_3$, Yield, 20% (60 mg). ^{1}H NMR (400 MHz, $CDCl_3$) δ: 4.50 (s, 1H), 3.10 (d, J = 6.3 Hz, 2H), 2.68 (t, J =

7.0 Hz, 2H), 1.35 (d, J = 73.5 Hz, 31H).

Compound **A3**. A solution of **A1** (200 mg, 0.53 mmol) and **A2** (158 mg, 0.53 mmol) in dry DMF (30 mL) was heated to 120°C under nitrogen atmosphere for 18 h. The resulting product was cooled to room temperature, washed with water and extracted with DCM. The organic layers were collected, dried over Na_2SO_4 and evaporated. The residue was purified by silica gel column with dichloromethane and ethanol (99:1). Yield, 45.4% (0.3 g). ^{1}H NMR (400 MHz, $CDCl_3$) δ: 8.76 (s, 4H), 4.52 (s, 1H), 4.29-4.10 (m, 4H), 3.10 (d, J = 6.4 Hz, 2H), 1.73 (dd, J = 15.0, 7.7 Hz, 4H), 1.44 (s, 9H), 1.43-1.10 (m, 28H), 0.88 (t, J = 6.8 Hz, 3H).

Compound **A4**. **A3** (200 mg, 0.302 mmol) was treated by trifluoroacetic acid (10 ml) and stirred at room temperature for 30 min. And then 10 ml of DCM was added and the mixture was washed with 5% aqueous solution of Na_2CO_3 to reach pH > 8. Organic layers were collected, dried over Na_2SO_4 and evaporated. Yield, 78% (120 mg). ^{1}H NMR (400 MHz, $CDCl_3$) δ: 8.76 (s, 4H), 4.28-4.10 (m, 4H), 2.68 (s, 2H), 1.80-1.69 (m, 4H), 1.47-1.22 (m, 28H), 0.91-0.78 (m, 3H).

Compound **A6**. According to the reported literatures,[3] **A5** (80 mg, 0.336 mmol) was dissolved in $CHCl_3$ (3 mL), After stirring for 10 min, the **A4** (200 mg, 0.356 mmol) soluble in 5 mL $CHCl_3$, and then was added dropwise. The mixture was stirred at room temperature for 12 h. the resulting mixture was added dropwise to hexane, and the produced solid was collected by centrifugation. The crude product was washed with THF at least twice. Yield, 77.3% (170 mg). ^{1}H NMR (400 MHz, $CDCl_3$) δ: 8.65 (s, 4H), 4.11 (s, 4H), 3.13 (s, 1H), 1.69 (s, 4H), 1.26 (s, 30H), 0.86 (s, 3H).

5.2 Synthesis of Semiconducting Polymer (C8-NDI)

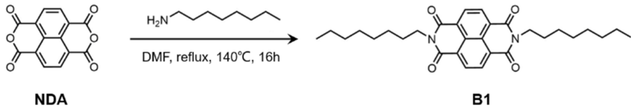

Supplementary Scheme 2. Synthesis process of semiconducting polymer (**C8-NDI**).

Compound **B1**. 1,4,5,8-Naphthalenetetracarboxylic dianhydride (1 g, 3.73 mmol) was added to a dry 100 mL flash. To the solid was added dry DMF (20 mL) and heated to

140°C under stirring in nitrogen atmosphere. After stirring for 10 min, the N-octylamine (0.964 g, 7.46 mmol) soluble in 10 ml dry DMF, and then was added dropwise. Cooling the reaction mixture in a refrigerator for 2 h. DMF was removed under reduced pressure and heated to 80°C. Added 50 ml of acetone to dissolve the mixture, cooled in a refrigerator overnight and the resulting precipitate was filtered. The filtrate was evaporated and the residue was purified by silica gel column with DCM. Yield, 80% (1.46 g). ^{1}H NMR (400 MHz, $CDCl_3$) δ: 8.76 (s, 4H), 4.30-4.10 (m, 4H), 1.79-1.67 (m, 4H), 1.48-1.19 (m, 20H), 0.88 (t, *J* = 6.6 Hz, 6H).

5.3 Synthesis of PolyAT and PolyES

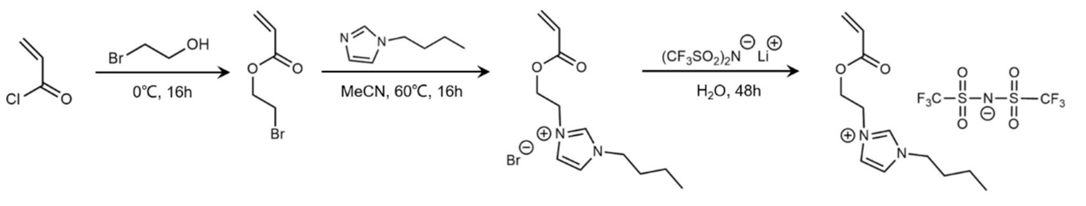

Supplementary Scheme 3. Synthesis process of **AT**.

Compound **C1.** 2-bromoethanol (7.09 mL, 0.1 mol) and CH_2Cl_2 (50 mL) were added to a 250 mL flask in an ice bath. After stirring for 10 min, triethylamine (13.9 mL) in 10 mL CH_2Cl_2 was added. Then acryloyl chloride (9.72 mL, 0.1 mol) dissolved in 10 mL CH_2Cl_2 was added dropwise through a constant pressure funnel. The mixture was vigorously stirred at room temperature for 16 h. The organic solution was collected by filtration and extracted thoroughly three times with deionized water. Then the solution was dried over anhydrous sodium sulfate and the solvent was removed by rotary evaporation to finally obtain a clear liquid product. Yield, 80% (17.9 g). 1H NMR (400 MHz, CDCl3) δ: 6.47 (dd, *J* = 17.3, 0.6 Hz, 1H), 6.16 (dd, *J* = 17.3, 10.5 Hz, 1H), 5.90 (dd, *J* = 10.4, 0.6 Hz, 1H), 4.48 (t, *J* = 6.1 Hz, 2H), 3.56 (t, *J* = 6.1 Hz, 2H).

Compound **C2.** According to the reported literatures, **C1** (1 g, 5.59 mmol) and 1-butylimidazole (0.73g, 5.86 mmol) were dissolved in acetonitrile (10 mL) and stirred at 60 °C under a nitrogen atmosphere for 16 h. After that, the solvent was removed by rotary evaporation. The product was dissolved in DI water and washed with CH_2Cl_2

three times.

Compound **C3** (**AT**)**:** bis(trifluoromethane)sulfonimide lithium salt (1.6 g, 5.59 mmol) was added to the **C2** aqueous solution for ion-exchange, and the mixture solution was stirred for 48 h at room temperature. Then, **AT** in the resulting solution was dissolved in CH_2Cl_2 and washed with DI water three times. The organic layer was dried with Na_2SO_4, and evaporated by rotary evaporation to afford **AT** as a slightly yellow transparent liquid. Yield, 40% (1.1 g). ^{1}H NMR (400 MHz, DMSO) δ: 9.24 (s, 1H), 8.12 – 7.63 (m, 2H), 6.34 (dd, J = 17.3, 1.4 Hz, 1H), 6.16 (dd, J = 17.3, 10.4 Hz, 1H), 6.00 (dd, J = 10.4, 1.3 Hz, 1H), 4.50 (td, J = 7.0, 4.2 Hz, 4H), 4.19 (t, J = 7.1 Hz, 2H), 1.87 – 1.66 (m, 2H), 1.22 (dd, J = 15.0, 7.4 Hz, 2H), 0.89 (t, J = 7.3 Hz, 3H). ^{19}F NMR (376 MHz, DMSO) δ: 78.76 (d, J = 2.9 Hz).

PolyAT Gel: AT (0.4 g, 0.8 mmol) and PEGDA (3.2 mg, 0.016 mmol) and 0.5 mol% of AIBN solution in ethanol (10 mg/mL) were vigorously mixed. Then, the ethanol was removed under vacuum at room temperature. The resulting viscous solution was injected between two glass slides (2 cm x 2 cm) separated by 0.3 mm thick plastic spacer, and then heated at 60 °C overnight in a N_2-filled glovebox. After the free radical polymerization was completed, the polyAT gel were washed with CH_2Cl_2 to remove unreacted monomers. Finally, the obtained gels were dried overnight in a vacuum oven at 60 °C.

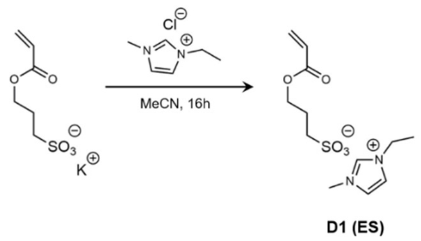

Supplementary Scheme 4. Synthesis process of **ES**.

Compound **D1 (ES):** According to the reported literatures,[4] 1-ethyl-3-methylimidazolium chloride (3.15 g, 21.5 mmol), 3-sulfopropyl acrylate potassium salt (5.0 g, 21.5 mmol) and methoxyphenol (5.0 mg, 0.04 mmol) were dissolved in acetonitrile (20 mL) and the mixture was vigorously stirred for 16 h at room

temperature. After the reaction, the by-product potassium chloride was removed by filtration, and the filtrate was concentrated and dried under vacuum. The resulting liquid was re-dissolved in CH_2Cl_2 and kept at < 0 °C overnight. The product was gained by filtered and concentrated to give **ES** as a viscous yellow oil. Yield, 50% (3.3 g). ^{1}H NMR (400 MHz, D_2O) δ: 8.58 (s, 1H), 7.35 (s, 1H), 7.28 (s, 1H), 6.31 (d, J = 17.3 Hz, 1H), 6.20 – 6.00 (m, 1H), 5.85 (d, J = 10.5 Hz, 1H), 4.17 (t, J = 6.2 Hz, 2H), 4.09 (q, J = 7.4 Hz, 2H), 3.75 (s, 3H), 2.98 – 2.82 (m, 2H), 2.13 – 1.89 (m, 2H), 1.36 (t, J = 7.4 Hz, 3H).

PolyES Gel: ES (0.4 g, 1.3 mmol) and PEGDA (5.3 mg, 0.026 mmol) and 107 μL AIBN solution in ethanol (10 mg/mL) were vigorously mixed. Then, the ethanol was removed by vacuum drying at room temperature. The viscous solution was injected between two glass slides (2 cm x 2 cm) separated by 0.3 mm thick plastic spacer, and then heated at 60 °C overnight in a N_2-filled glovebox. After the free radical polymerization was completed, the polyES gel were washed with CH_2Cl_2 to remove unreacted monomers. Finally, the obtained gels were dried overnight in a vacuum oven at 60 °C.

PolyAT Gel and PolyES Gel: AT (or **ES**) (0.4 g) and 2 mol% purified PEGDA and 0.5 mol% of AIBN solution in ethanol (10 mg/mL) were vigorously mixed. Then, the ethanol was removed under vacuum at room temperature. The resulting viscous solution was injected between two glass slides (2 cm x 2 cm) separated by 0.3 mm thick plastic spacer, and then heated at 60 °C overnight in a N_2-filled glovebox. After the free radical polymerization was completed, the **PolyAT** (or **PolyES**) **Gel** were washed with CH_2Cl_2 to remove unreacted monomers. Finally, the obtained gels were dried overnight in a vacuum oven at 60 °C.

5.4 NMR Spectra

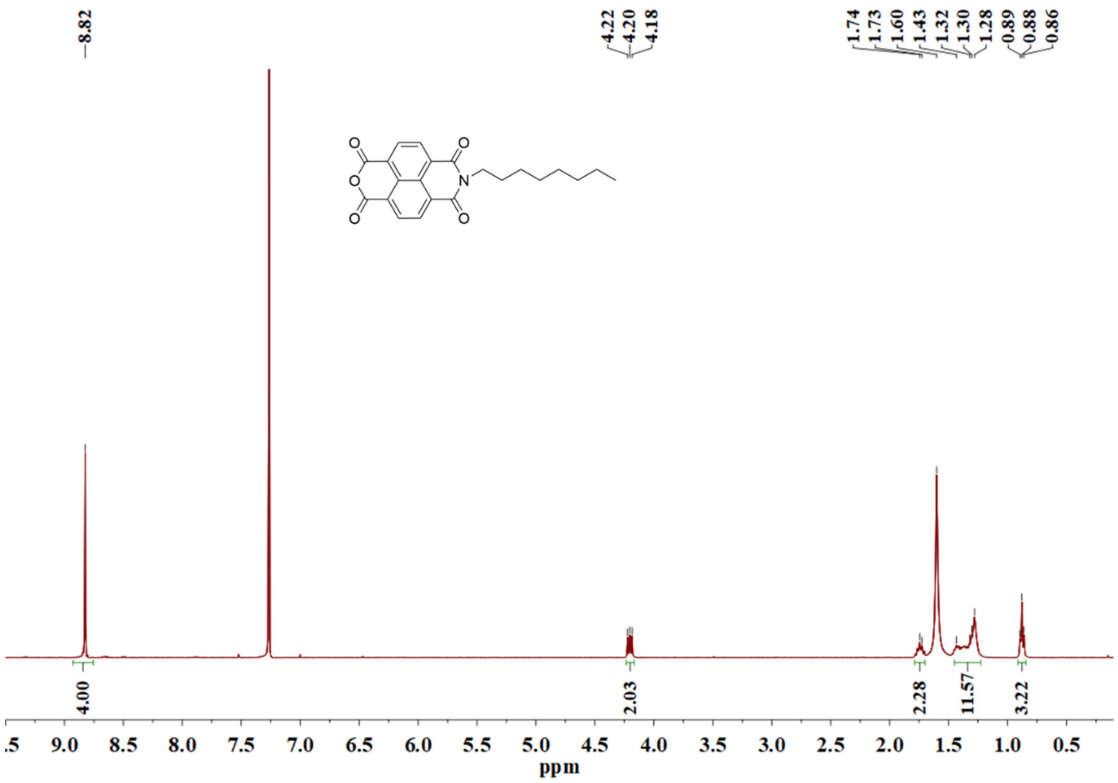

Supplementary Fig. 34 | The 1H NMR spectrum (400 MHz) of **A1** in $CDCl_3$.

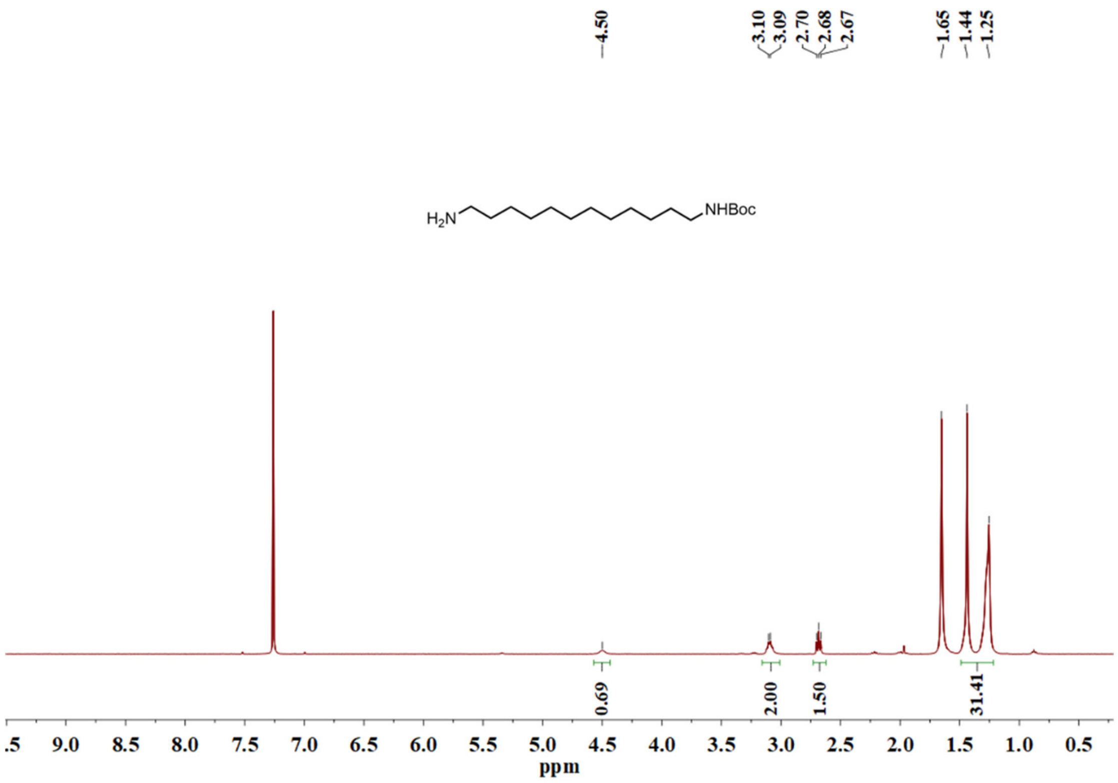

Supplementary Fig. 35 | The 1H NMR spectrum (400 MHz) of **A2** in $CDCl_3$.

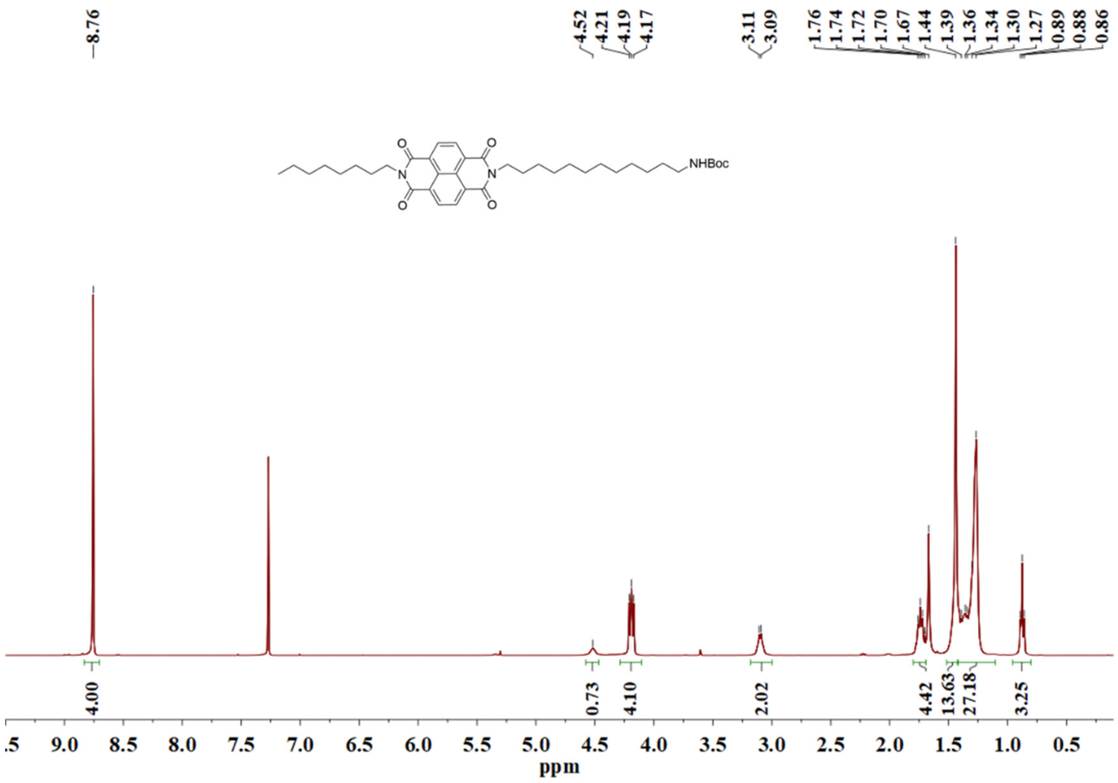

Supplementary Fig. 36 | The ^{1}H NMR spectrum (400 MHz) of **A3** in $CDCl_3$.

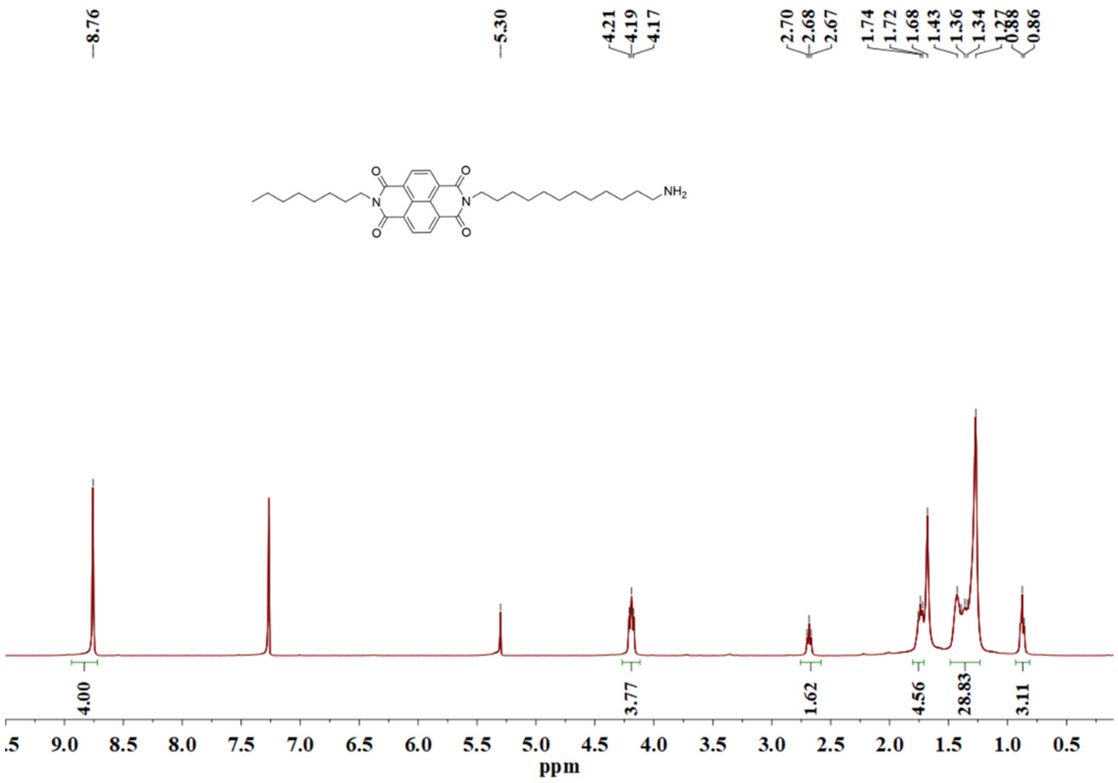

Supplementary Fig. 37 | The ^{1}H NMR spectrum (400 MHz) of **A4** in $CDCl_3$.

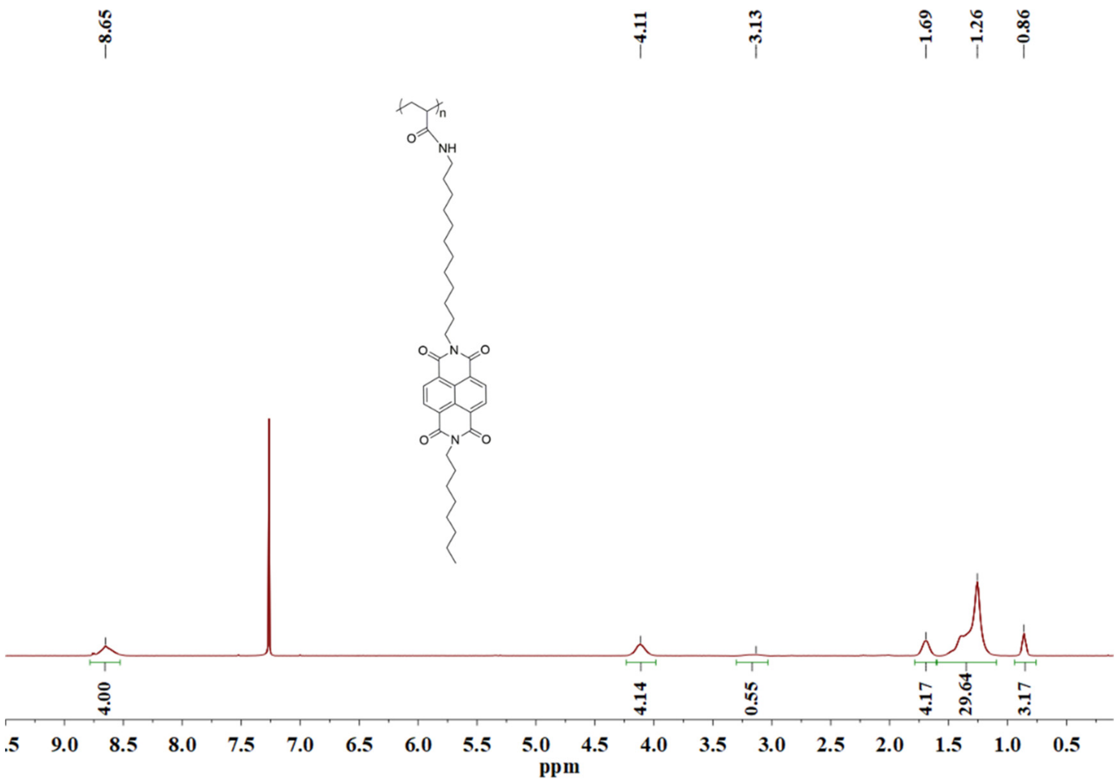

Supplementary Fig. 38 | The ^{1}H NMR spectrum (400 MHz) of ***p*-NDI** in $CDCl_3$.

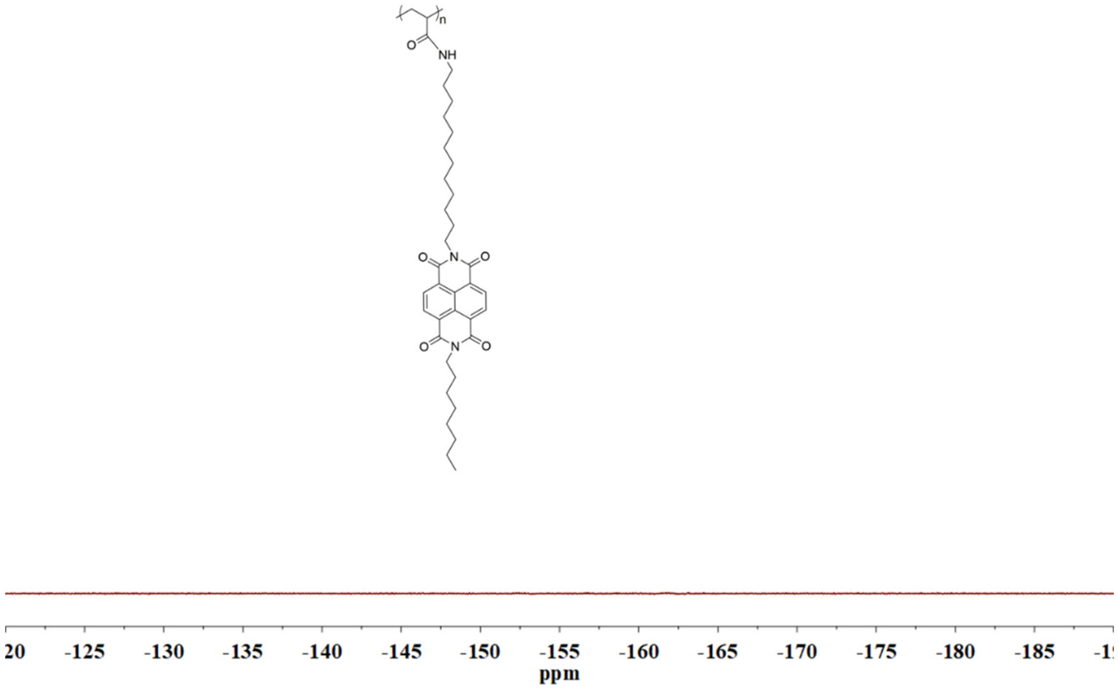

Supplementary Fig. 39 | The ^{19}F NMR spectrum (400 MHz) of ***p*-NDI** in $CDCl_3$.

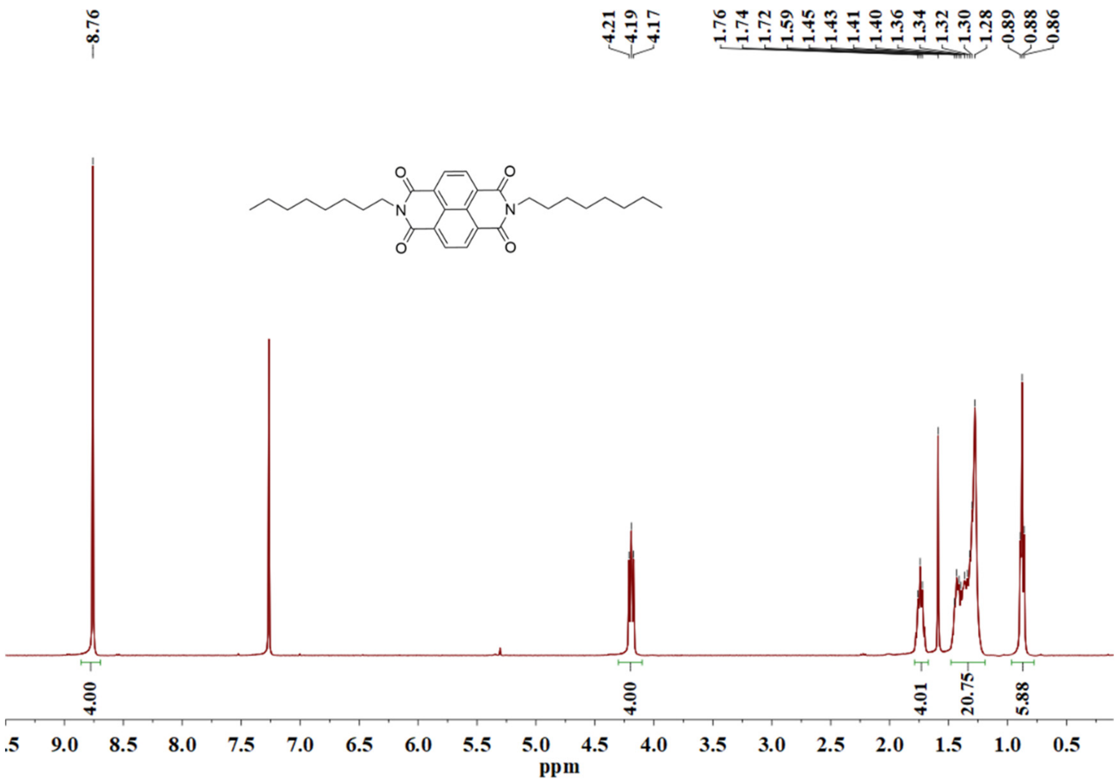

Supplementary Fig. 40 | The ^{1}H NMR spectrum (400 MHz) of **C8-NDI** in $CDCl_3$.

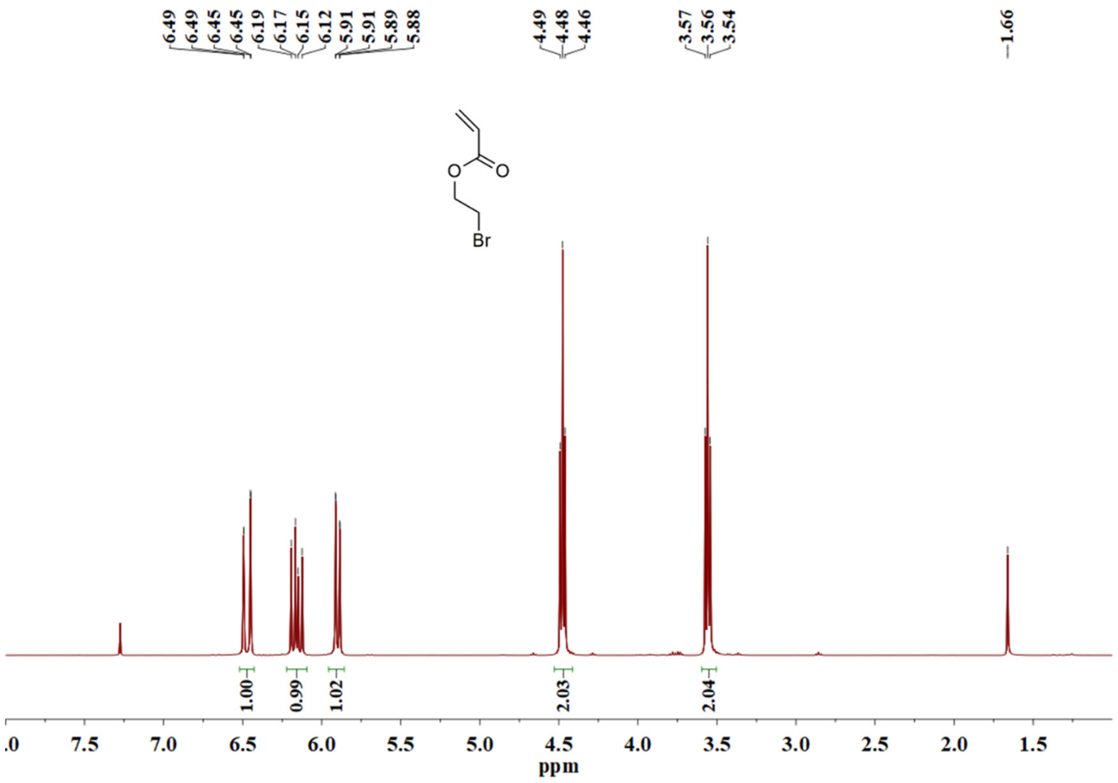

Supplementary Fig. 41 | The ^{1}H NMR spectrum (400 MHz) of **C1** in $CDCl_3$.

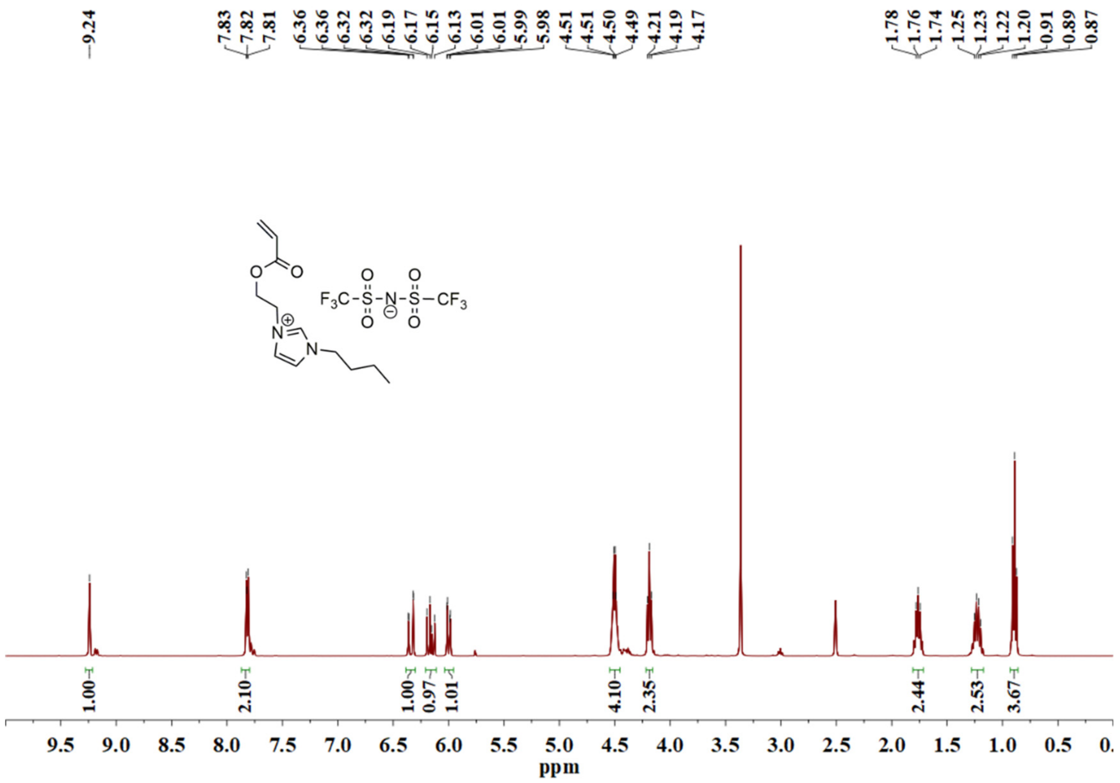

Supplementary Fig. 42 | The ^{1}H NMR spectrum (400 MHz) of **AT** in d$_6$-DMSO.

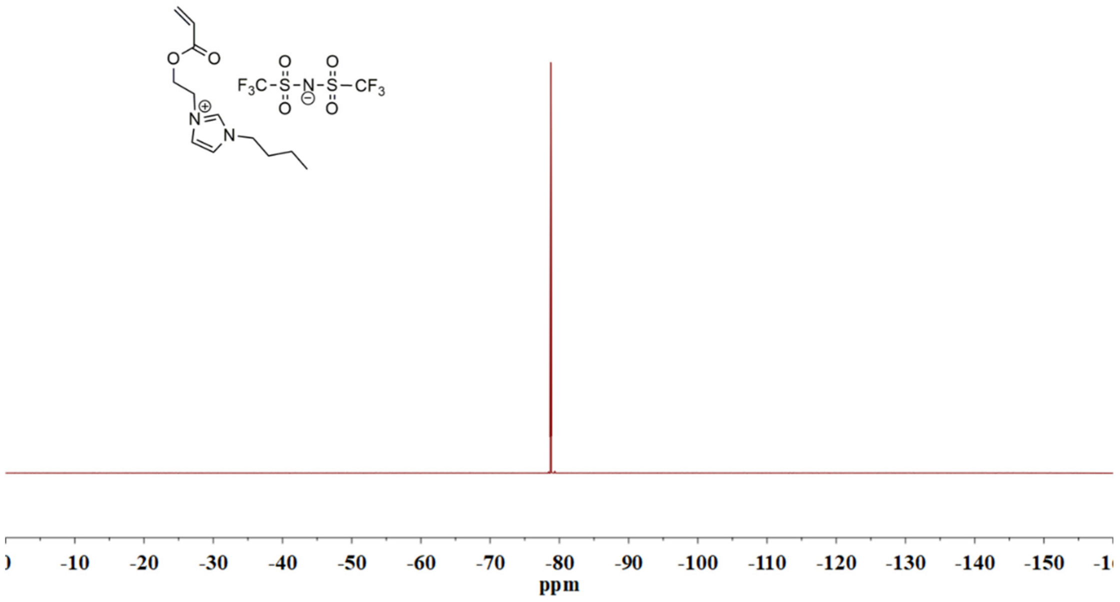

Supplementary Fig. 43 | The ^{19}F NMR spectrum (400 MHz) of **AT** in d$_6$-DMSO.

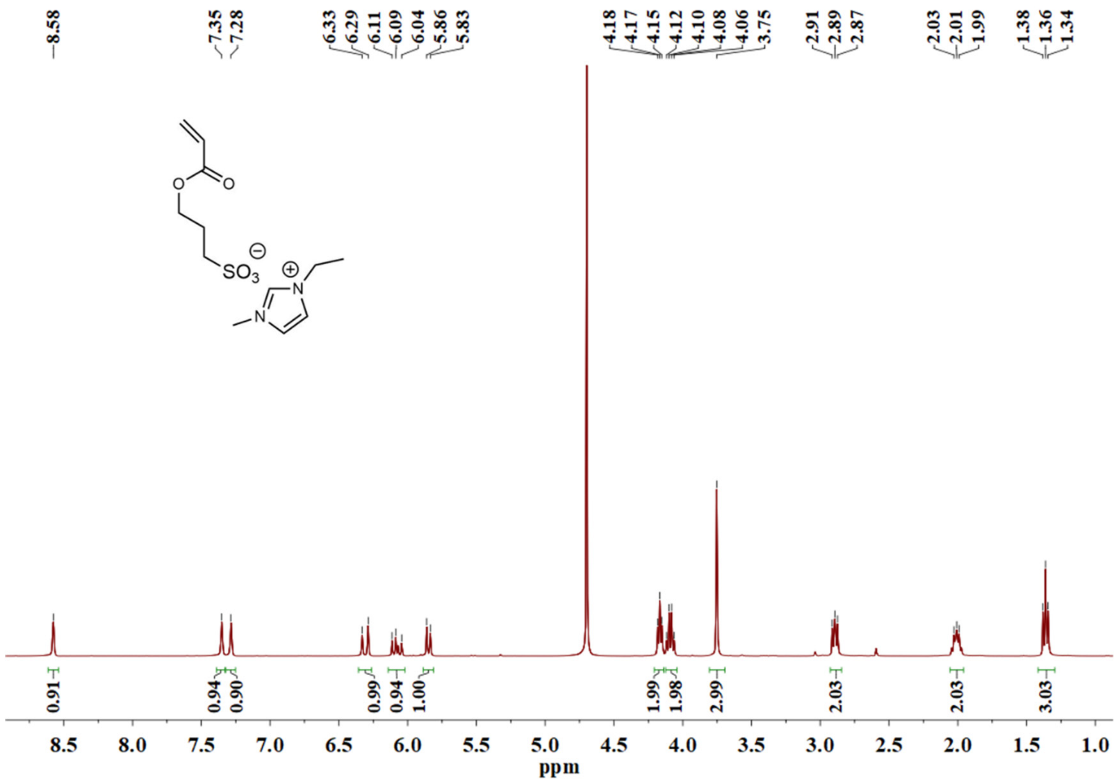

Supplementary Fig. 44 | The ¹H NMR spectrum (400 MHz) of **ES** in D_2O.

Supplementary references:

[1] S. Milita, F. Liscio, L. Cowen, M. Cavallini, B. A. Drain, T. Degousée, S. Luong, O. Fenwick, A. Guagliardi, B. C. Schroeder, N. Masciocchi, *J. Mater. Chem. C 8*, 3097-3112 (**2020**).

[2] A. Morrell, M. S. Placzek, J. D. Steffen, S. Antony, K. Agama, Y. Pommier, M. Cushman, *J. Med. Chem. 9*, 2040-2048 (**2007**).

[3] X. Wu, S. Feng, J. Shen, W. Huang, C. Li, C. Li, Y. Sui, W. Huang, *Chem. Mater. 32*, 3641-3650 (**2020**).

[4] H. Chen, J.-H. Choi, D. Salas-de la Cruz, K. I. Winey, Y. A. Elabd, *Macromolecules 42*, 4809-4816 (**2009**).
