# Supplementary material for: Wearable in-sensor reservoir computing using optoelectronic polymers with through-space charge-transport characteristics for multi-task learning
Source: Nat Commun. 2023 Jan 28;14:468. doi: 10.1038/s41467-023-36205-9 (PMC9884246; doi:10.1038/s41467-023-36205-9)
Supplement: Supplementary file 3 — Description of Additional Supplementary Files [file 41467_2023_36205_MOESM3_ESM.docx]

**Description of Additional Supplementary Files**

**File Name: Supplementary Movie 1
Description:** Hand clapping.

**File Name: Supplementary Movie 2
Description:** Left-hand waving.

**File Name: Supplementary Movie 3
Description:** Right-hand waving.
